# Activating in-plane dead zones of anode catalyst layers in proton exchange membrane water electrolyzers

**DOI:** 10.1038/s41467-026-75223-1

**Published:** 2026-07-01

**Authors:** Zicheng Zhao, Xiao Liang, Nan Lin, Zhenye Kang, Yuchang Hou, Yaoxin Wang, Hui Chen, Xiaoxin Zou

**Affiliations:** 1 State Key Laboratory of Inorganic Synthesis and Preparative Chemistry, College of Chemistry, Jilin University, Changchun, China; 2 Key Laboratory of Agro-Forestry Environmental Processes and Ecological Regulation of Hainan Province, State Key Laboratory of Tropic Ocean Engineering Materials and Materials Evaluation, Hainan Provincial Key Lab of Fine Chem, School of Environmental Science and Engineering, Hainan University, Haikou, China; 3 Changbaishan Laboratory, Changchun, China; 4 National Demonstration Center for Experimental Chemistry Education, Jilin University, Changchun, China

**Keywords:** Electrocatalysis, Electrocatalysis, Electrocatalysis, Hydrogen energy

## Abstract

The utilization of iridium-based anode catalysts in proton exchange membrane water electrolyzers is largely limited by the presence of electrochemically inactive “dead zones” within the catalyst layer. Here, by combining in-situ visualization of gas bubble evolution with quantitative conductivity measurements and electrochemical analysis, we establish that the limited in-plane electronic conductivity, dictated by the ionomer disrupting the conductive network of IrO_2_ nanocatalysts, is the dominant factor. A sequential spray-coating strategy is further developed, which decouples the deposition of a pristine conductive catalyst layer from the ionomer and thereby preserves continuous electron transport pathways. This approach effectively activates the in-plane dead zones, resulting in membrane electrode assemblies that exhibit over 30% higher activity (4.2 A cm^−2^@2.0 V@80 °C; membrane: Nafion 115) than those prepared by the conventional one-step method based on IrO_2_/ionomer mixed inks. Crucially, this approach achieves both low iridium loading (0.25 mg cm^–2^) with standard catalysts and demonstrates extended stability over 8300 hours under practical current densities.

## Abstract (web summary)

Anode catalyst layers in proton exchange membrane water electrolyzers contain electrochemically inactive zones that limit iridium utilization. Here, the authors identify ionomer-induced conductivity loss as the root cause and develop a sequential spray-coating strategy that activates these zones.

## Introduction

**Fig. 1:**
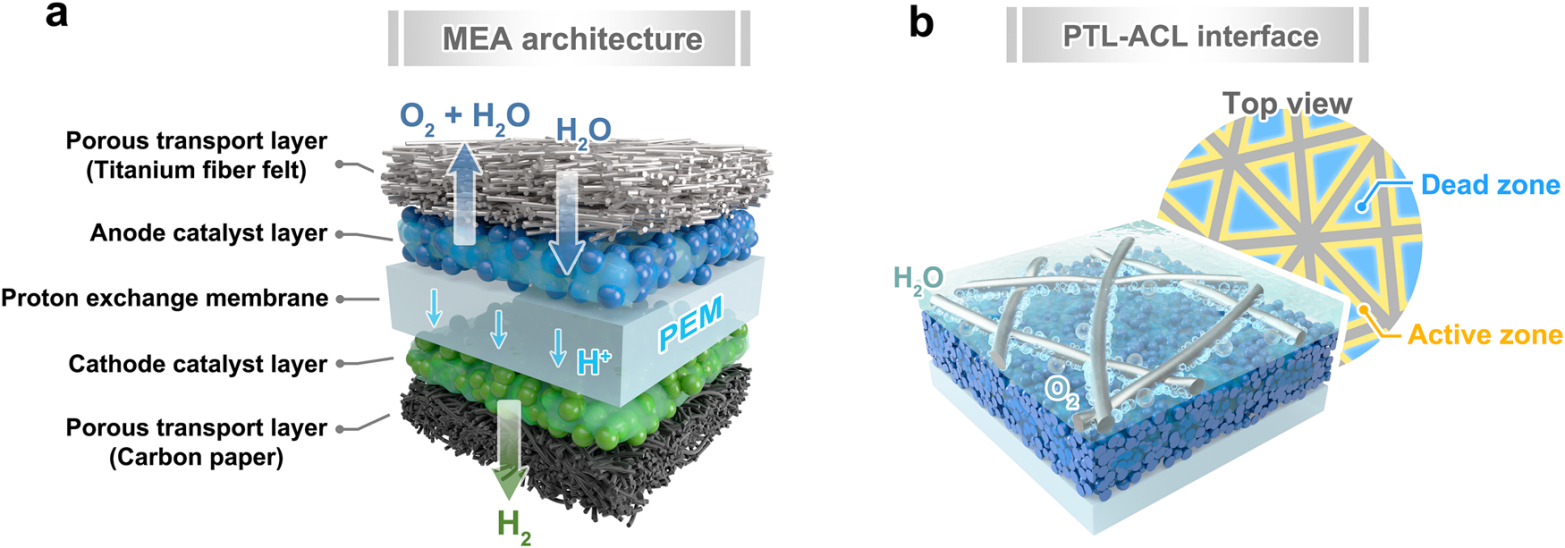
Schematic diagram of membrane electrode assembly architecture and electrochemically active distribution. **a** Schematic illustration of the architecture and working principle of a membrane electrode assembly (MEA) in PEMWE. **b** Schematic of the interface between the porous transport layer (PTL) and the anode catalyst layer (ACL), depicting the distribution of electrochemically active and inactive (“dead”) zones across the ACL.

The global imperative for decarbonization has elevated green hydrogen to a critical energy vector^1^. Proton exchange membrane water electrolysis (PEMWE) stands out among hydrogen production technologies due to its high efficiency, rapid response to renewable power variations, and compact design^2–5^. Central to the PEMWE is the membrane electrode assembly (MEA), which integrates the catalyst layers, proton exchange membrane (PEM), and porous transport layers (PTLs) (Fig. 1a). This integrated structure is responsible for all electrochemical reactions, and its performance ultimately determines the electrolyzer’s efficiency, lifetime, and cost ceiling^6–9^. The current mainstream approach is based on the catalyst-coated membrane (CCM) structure, where the CCM is positioned between two PTLs. The CCM features catalyst layers that are directly applied onto the PEM, with Pt- and Ir-based materials catalyzing the hydrogen evolution and oxygen evolution reactions at the cathode and anode, respectively^10–14^. This design not only enables precise control over catalyst distribution and superior interfacial contact but also lends itself to scalable, continuous manufacturing processes. Nevertheless, the utilization of the Ir-based anode catalyst remains intrinsically low, typically below 30%^15,16^. This issue was visually presented in 2016 by Mo et al.^17^, who employed an optically accessible electrochemical cell and revealed a highly non-uniform distribution of electrochemical activity within the anode catalyst layer (ACL). Their work identified electrochemically inactive “dead zones” located in regions far from the PTL (Fig. 1b), where Ir-based nanocatalysts do not participate in the catalytic reaction. This observation provides critical evidence that the low catalyst utilization originates from fundamental transport limitations rather than from the catalyst itself, which leads to significant portions of the ACL being electrochemically inaccessible under operating conditions.

In pursuit of higher Ir utilization, this insight has stimulated the exploration of alternative MEA fabrication strategies, including the catalyst-coated substrate approach^18–21^, in which the catalyst is directly deposited onto the PTLs. In essence, this configuration avoids placing any catalyst in the regions that would otherwise become “dead zones” in a conventional CCM. Despite considerable advances in reducing Ir loading and improving utilization efficiency^22–26^, the CCM method continues to dominate industrial practice owing to its overall manufacturability and integration advantages. Within the CCM framework, however, the fundamental origin of the dead zones remains ambiguous. Although poor electronic conductivity in the ACL has been implicated^27–29^, the quantitative link between conductivity and catalytic efficacy has not been established, and the main factors governing conductivity are still inadequately understood. The development of effective approaches to activate these inactive regions thus represents a critical and unresolved challenge.

In this study, by combining in-situ visualization of gas bubble evolution with conductivity measurements and electrochemical analysis, we establish a quantitative correlation between the extent of catalytic activity in so-called “dead zones” and electronic conductivity, and find that the limited electronic conductivity is primarily due to the insulating ionomers that disrupt the conductive network of Ir-based nanocatalysts. Furthermore, we introduce a sequential spray-coating approach, in which a pristine, conductive catalyst layer is first deposited on the PEM, followed by the application of a separate ionomer overlayer. This architecture maintains continuous electron transport pathways across the catalyst layer and effectively activates the in-plane dead zones. The resulting MEAs show a great enhancement in electrochemical activity, outperforming conventional one-step spray-coated electrodes by over 30%. Our study provides the critical experimental demonstration that the inactive regions within the catalyst layer can be effectively activated.

## Results and discussion

**Fig. 2:**
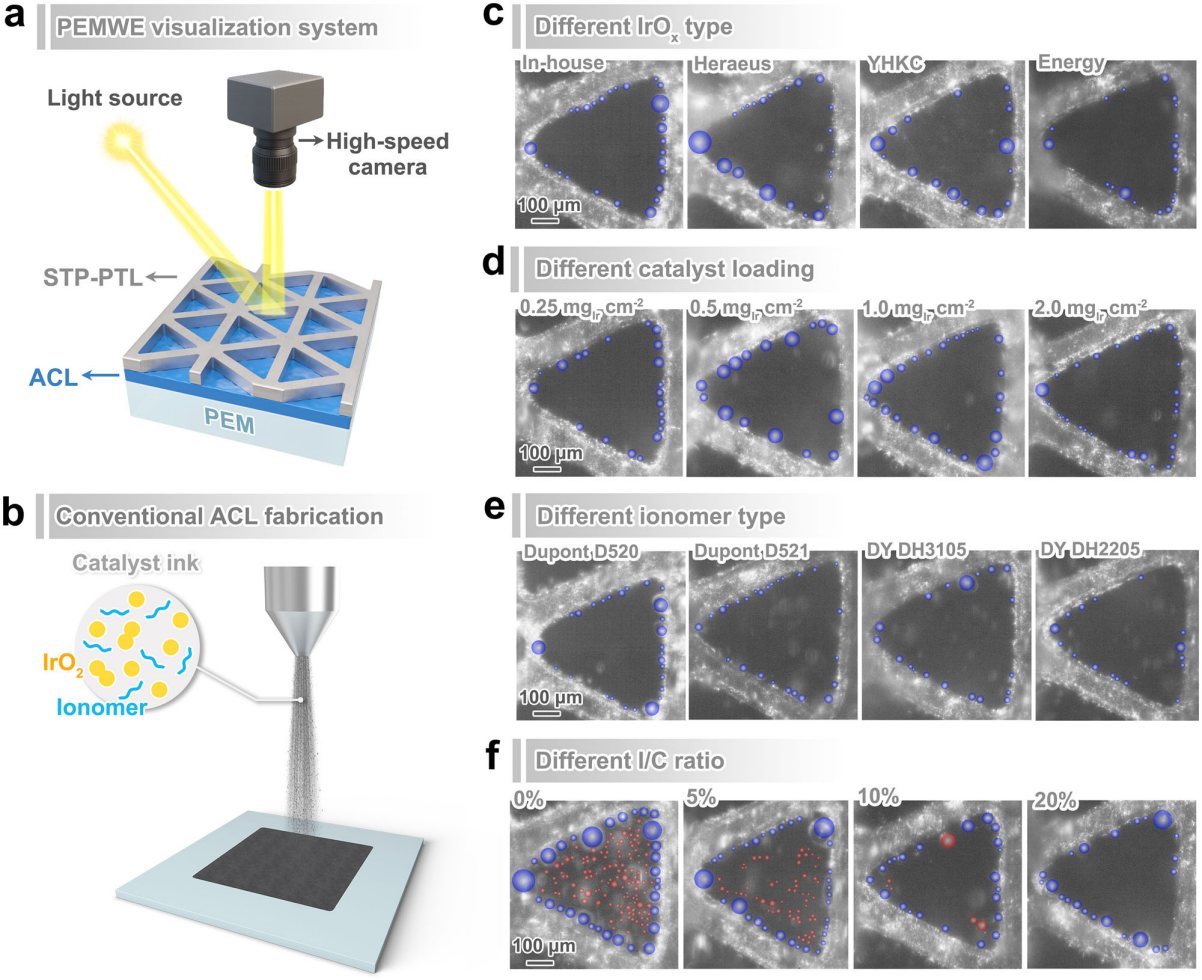
In-situ visualization of gas bubble evolution in the catalyst layer. **a** Schematic of a transparent and reaction-visible PEMWE equipped with a high-speed and microscale visualization system. **b** Schematic of conventional ACL fabricated by spray-coating a homogeneous ink of IrO_2_ catalyst nanoparticles and ionomer. **c**–**f** Front-view visualization of gas bubble evolution occurring within the microchannel of PEMWEs employing different catalyst types, catalyst loadings, ionomer types, and ionomer/catalyst ratios.

Gas bubble evolution during electrochemical reaction in an operating PEMWE was investigated using a self‑built visualization system comprising two key components (Fig. 2a and Supplementary Fig. 1). The first is a transparent, reaction-visible cell that integrates a thin Ti-based PTL with straight-through pores (STP-PTL, Supplementary Fig. 2), enabling direct optical access to the ACL. The second is a high-speed, microscale visualization system capable of capturing the real‑time spatial distribution of bubble nucleation sites within the ACL. The spatial distribution of bubble nucleation sites as a proxy for the distribution of electrochemically active regions. This integrated setup was used to probe the origin of electrochemically inactive zones in a conventional ACL, which is fabricated by spray-coating a homogeneous ink of IrO_2_ catalyst nanoparticles and ionomer (Fig. 2b). To systematically elucidate the governing factors, the catalyst type, catalyst loading, ionomer type, ionomer/catalyst ratio, and operating conditions were varied independently.

To assess the effect of catalyst type on reaction zone formation, four conventional ACLs were prepared using different IrO_2_ nanocatalysts, including in-house synthesized nanoparticles (Supplementary Fig. 3) and three commercial nanocatalysts from Heraeus (Germany, Supplementary Fig. 4), YHKC (China, Supplementary Fig. 5), and Energy Chemical (China, Supplementary Fig. 6). All ACLs are fabricated with a fixed iridium loading of 0.25 mg_Ir_ cm^−2^ and an ionomer/catalyst ratio of 15 wt% (DuPont D520), which lies within the typical ionomer range optimized for balanced activity and stability in commercial applications^30–35^. Results from the in-situ visualization system (Fig. 2c and Supplementary Video 1) reveal that, regardless of the catalyst type, oxygen bubbles are generated exclusively within a narrow zone of the catalyst layer adjacent to the PTL, with no bubble formation observed in the middle region of openings. Furthermore, systematic variations in catalyst loadings (Fig. 2d and Supplementary Video 2, 0.25–2.0 mg cm^−2^), ionomer types (Fig. 2e and Supplementary Video 3, DuPont D520, DuPont D521, DY DH3105, and DY DH2205), current density (Supplementary Fig. 7 and Video 4, 0.1–2.0 A cm^−2^), and clamping pressure (Supplementary Video 5, 1–7 N m bolt torque) all yield the same pattern that bubble nucleation sites remain confined to the boundary region of the PTL and the ACL. These collective results demonstrate that the formation of in-plane dead zones is a universal phenomenon, independent of catalyst type, catalyst loading, ionomer type, or operating conditions.

**Fig. 3:**
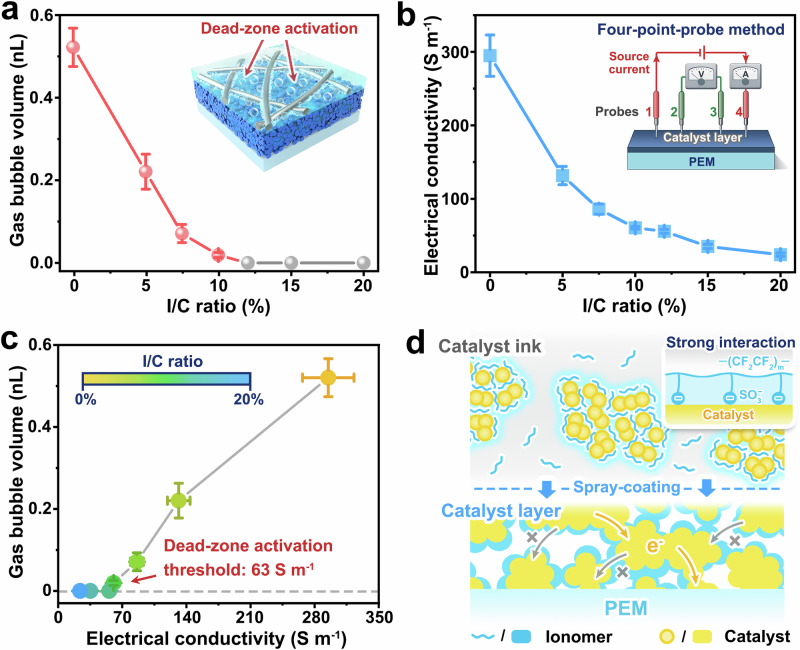
The correlation among I/C ratio, electronic conductivity of the catalyst layer, and in-plane electrochemical activity. **a** Correlation between gas bubble volume within a single PTL opening (middle region, 40 ms) and I/C ratio. The inset illustrates the activation of the electrochemically dead region within the ACL when the I/C ratio is ≤10 wt%. **b** Dependence of the in-plane electronic conductivity of the catalyst layer on I/C ratio. The inset shows a schematic of the four-point probe method used to measure the electronic conductivity of the ACL. **c** Correlation between the gas bubble volume within a single PTL opening (middle region, 40 ms) and the in-plane electronic conductivity of the catalyst layer, revealing a critical conductivity threshold required for in-plane electrochemical activation. Note that this threshold depends on the PTL geometry and may shift with different PTL structures. **d** Schematic illustration of the ionomer-induced disruption of the conductive network of IrO_2_ nanocatalysts.

Next, the ionomer content was varied from 0 to 20 wt% to isolate its role, using ACLs fabricated with in-house synthesized IrO_2_ at a constant iridium loading of 0.25 mg_Ir_ cm^−2^ (Fig. 2f, Supplementary Fig. 8 and Video 6). In-situ gas bubble evolution visualization clearly shows that bubble formation depends on the ionomer/catalyst ratio. At levels above 10 wt%, it remains strictly confined to the edge of the PTL. When the ionomer/catalyst ratio is reduced to 10 wt%, bubble nucleation sites begin to emerge in the middle of the openings of the PTL. The population of bubbles in this middle region increases progressively with further reduction of ionomer/catalyst ratio, with the absence of ionomer leading to the most vigorous gas evolution in the middle zone. To quantify this effect, the gas volume generated in the middle of the openings of the PTL was statistically analyzed and correlated with the ionomer/catalyst ratio. As shown in Fig. 3a, when the ionomer/catalyst ratio is ≤10 wt%, the gas bubble volume in the middle region increased in an approximately linear manner with decreasing ionomer content. This trend demonstrates the progressive activation of the electrochemically dead region within the ACL. These results demonstrate that the ionomer content dictates whether the reaction occurs solely at the PTL edge or extends across the catalyst layer.

Given the ionomer’s decisive role in spatially confining the reaction, understanding its mechanism becomes critical. A prevailing hypothesis attributes the formation of dead zones to insufficient electronic conductivity within the catalyst layer^27–29^. This notion, however, has remained a qualitative speculation lacking direct quantitative experimental validation. To establish this crucial relationship, the in-plane electronic conductivity of the ACL was systematically measured and correlated with the ionomer/catalyst ratio (Fig. 3b and Supplementary Fig. 9). Using a four-point probe method, conductivity was found to increase monotonically as the ionomer content decreased. Specifically, reducing the ionomer/catalyst ratio from 20 wt% to 0 yields an approximately 12-fold enhancement in conductivity. This quantitative evidence confirms that the ionomer is a critical governing factor of the electronic conductivity within the catalyst layer.

A direct relationship between the electronic conductivity of the ACL and its in-plane electrochemical activity was further established (Fig. 3c). Conductivity was tuned by varying the ionomer/catalyst ratio, while the corresponding in-plane activity was quantified by the gas volume generated in the middle of the openings of PTL. A strong positive correlation was observed, revealing a critical activation threshold at approximately 63 S m^−1^. Beyond this threshold, the gas bubble volume in the central region increased steadily with conductivity as the ionomer/catalyst ratio was reduced from 20 wt% to 0. These results, therefore, establish that the formation of an electrochemical dead zone can be attributed directly to the ionomer-induced limitation of in-plane electronic conductivity.

To elucidate the underlying mechanism of the ionomer effect, the uniformly mixed ionomer-IrO_2_ catalyst ink was first investigated. Dynamic light scattering (DLS) measurements (Supplementary Fig. 10) indicate the formation of agglomerates with an average diameter of approximately 400 nm in the ink state. Transmission electron microscopy (TEM, Supplementary Fig. 11a) of the dried ink reveals dense IrO_2_ agglomerates ranging from ~100 nm to 1 μm in size. Elemental mapping (Supplementary Fig. 11b) shows a homogeneous nanoscale distribution of fluorine (a signature element of ionomer) throughout these agglomerates, confirming strong interactions between IrO_2_ nanoparticles and the ionomer.

To gain molecular-level insight into this aggregation process, molecular dynamics simulations were performed on systems containing ionomer and IrO_2_ clusters in a water/isopropanol mixed solvent. The simulations (Supplementary Fig. 12 and Supplementary Data 1) reveal that the interaction between IrO_2_ clusters and the sulfonate (-SO_3_H) groups of the ionomer side chains drives the formation of the observed dense IrO_2_/ionomer agglomerates (Fig. 3d above).

Further evidence from scanning electron microscopy (SEM, Supplementary Fig. 13) of the fabricated catalyst layer confirms that this aggregated structure is preserved, with the layer being composed of IrO_2_-ionomer agglomerates on a scale of several hundred nanometers. Consequently, the continuous conductive network of IrO_2_ nanoparticles is disrupted by the insulated ionomers (Fig. 3d below and Supplementary Fig. 14), which fundamentally limits the in-plane electronic conductivity of the catalyst layer.

**Fig. 4:**
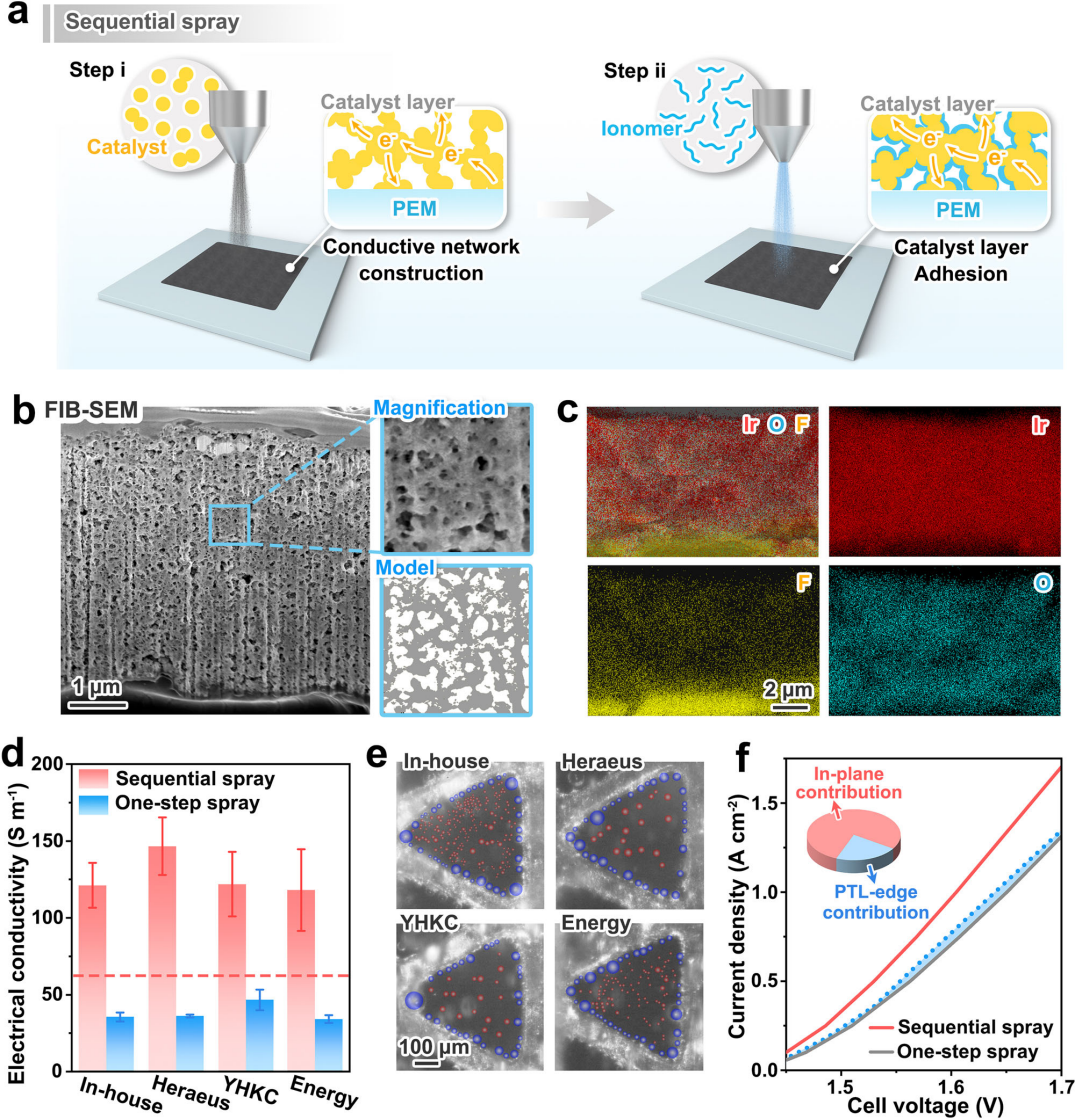
Construction of sequentially sprayed catalyst layers with enhanced in-plane conductivity. **a** Schematic illustration of the sequential spray fabrication strategy for constructing the catalytic layer. **b** Focused ion beam SEM image and reconstructed 2D model of the catalytic layer fabricated via the sequential spray strategy. **c** Elemental mapping images of Ir, O, and F in the sequentially sprayed layer. **d** In-plane electronic conductivities of sequentially sprayed layers and one-step sprayed layers based on four different IrO_2_ catalysts. The horizontal dashed line indicates the 63 S m^−1^ threshold required for in-plane activation. **e** Front-view visualization of electrochemical reactions occurring within the microchannel of PEMWEs employing different sequentially sprayed layers at a current density of 2 A cm^−2^. **f** Activity analysis quantifying the edge and in-plane contributions for cells with sequentially sprayed and one-step sprayed layers.

Inspired by these findings, we proposed a sequential spray fabrication strategy to resolve the conflicting roles of the ionomer as both an essential binder and a proton conductor (Supplementary Figs. 15 and 16), which otherwise compromises electronic conductivity. As illustrated in Fig. 4a, this approach decouples these functions through two distinct steps: first, spray-depositing an ionomer-free IrO_2_ ink to form a continuous, three-dimensional conductive skeleton; then, infiltrating ionomer into this pre‑formed network to provide binding and proton conduction. This architecture maintains mechanical integrity and ionic pathways while effectively preserving the in-plane electron transport network.

Four catalyst layers were fabricated using the sequential spray approach, based on different IrO_2_ sources, including an in-house synthesized material and three commercial catalysts from Heraeus, YHKC, and Energy Chemical. Using the in-house synthesized IrO_2_ as a representative example, characterizations of the ionomer-free ink (Supplementary Fig. 17) and the resulting catalyst layer (Fig. 4b and Supplementary Fig. 18) confirm that the IrO_2_ nanoparticles assemble into an interconnected, three-dimensional framework. Furthermore, elemental mapping (Fig. 4c) shows a uniform distribution of fluorine throughout the layer, which indicates successful infiltration of the ionomer into this pre‑constructed structure.

The in-plane electronic conductivity of all catalyst layers prepared by the sequential spray process was quantified using a four-point probe method^36^, with the corresponding conventionally prepared one-step catalyst layers serving as benchmarks. As shown in Fig. 4d, the sequential spray layers exhibit conductivities in the range of 120–150 S m^−1^ (Supplementary Fig. 19). This represents a three to four times enhancement over the conventional one-step layers (30–50 S m^−1^) and exceeds the 63 S m^−1^ threshold required for in-plane activation. Consistent with this, in-situ visualization shows pronounced bubble formation across the central region of the PTL for the sequential spray layers (Fig. 4e and Supplementary Video 7), in stark contrast to the conventional layers where reaction remains confined to the PTL edge (Fig. 2c). These results demonstrate that the sequential spray approach effectively activates the in-plane dead zones. This is achieved by creating a continuous conductive framework that coexists with the ionomer network required for structural integrity and proton transport.

Based on the in-situ visualization results, the system was further used to quantitatively decouple the activity contributions from the edge and the central region of the PTL (Fig. 4f). Electrolyzers employing the sequentially sprayed catalyst layers demonstrate significantly higher overall activity than those using conventional one-step sprayed catalyst layers. For example, at a cell voltage of 1.6 V, the sequentially sprayed catalyst layer achieves a current density of 1.0 A cm^−2^, representing a 40% increase over the conventional catalyst layer prepared by one-step spraying of a pre-mixed ionomer/IrO_2_ ink (0.7 A cm^−2^).

In contrast to the conventional catalyst layers, where activity is confined to the PTL edge, the sequentially sprayed layers exhibit substantial contributions from both the edge and the central region. By statistically analyzing the bubble volumes generated in these two zones, their respective activity contributions were quantitatively separated. Compared with the conventional layers, approximately 77% of the activity enhancement in the sequentially sprayed layer arises from the activation of the in‑plane dead zones, while the remaining 23% is attributed to improved activity at the edge. This work enables a quantitative decoupling of edge and in‑plane activity contributions in a PEM water electrolyzer, which was previously inaccessible experimentally.

**Fig. 5:**
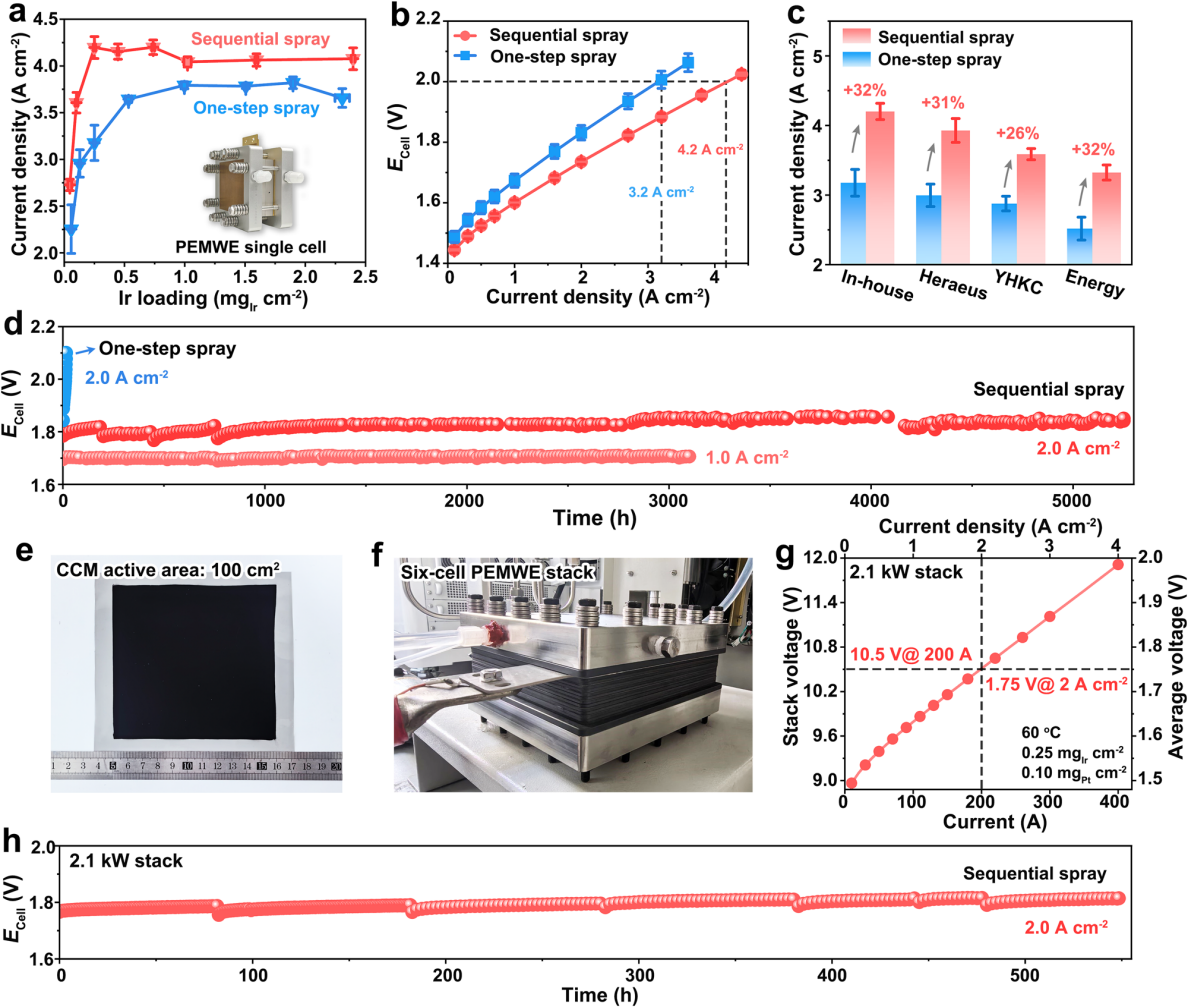
Performance assessment of PEMWEs with sequentially sprayed anodes from a single cell to the stack level. **a** Comparison of PEMWE performance using sequentially sprayed layer and one-step sprayed layer as anodes with different Ir loadings (80 °C, Nafion 115, 2.0 V). The inset shows a photograph of the PEMWE cell. **b** Polarization curves of PEMWEs without iR-corrected using sequentially sprayed layer and one-step sprayed layer as anodes at Ir loadings of 0.25 mg_Ir_ cm^−2^. **c** Comparison of PEMWE performance using sequentially sprayed layer and one-step sprayed layer as anodes with different catalyst types at Ir loadings of 0.25 mg_Ir_ cm^−2^ (80 °C, Nafion 115, 2.0 V). **d** Chronopotentiometry curves of PEMWEs using sequentially sprayed layer and one-step sprayed layer as anodes at ampere-level current densities. **e** Photograph of a large-area CCM with an active area of 100 cm^2^. **f** Photograph of a 2.1 kW PEMWE stack consisting of six cells. **g** Steady-state polarization curve for the PEMWE stack with sequentially sprayed layer. **h** Chronopotentiometric stability curve of sequentially sprayed layer-based PEM stack at a current density of 2 A cm^−2^.

Given its ability to effectively activate previously inactive regions within the catalyst layer, the sequentially sprayed catalyst layer is expected to serve as an efficient anode in practical PEM water electrolysis. A full PEMWE single cell was assembled using commercial titanium fiber felt as the PTL, and Nafion 115 as the PEM (5 cm^2^). High-frequency resistance (HFR) derived from electrochemical impedance spectroscopy (EIS, Supplementary Fig. 20) confirmed the enhanced electronic connectivity established by the sequential spray strategy. The effect of Ir catalyst loading on PEMWE performance was investigated using in-house-synthesized IrO_2_ at 80 °C (Fig. 5a and Supplementary Fig. 21). For both the sequentially sprayed layer and the conventional sprayed catalyst layer, the cell performance initially increases with increasing Ir loading and eventually reaches a plateau, indicating saturation of electrochemically accessible active sites. In the case of a conventional sprayed layer, a stable activity is achieved at a high Ir loading of 1.0 mg_Ir_ cm^−2^, delivering a current density of 3.8 A cm^−2^ at 2.0 V. In contrast, the sequentially sprayed layer reaches a stable and higher activity at a much lower Ir loading of 0.25 mg_Ir_ cm^−2^, achieving a current density of 4.2 A cm^−2^ at 2.0 V, which exceeds the maximum activity attainable by the conventional sprayed layer.

We evaluated the performance of PEMWEs using sequentially sprayed layers and one-step sprayed layers as the anodes, both at the same low Ir loading of 0.25 mg_Ir_ cm^−2^. Figure 5b presents the polarization curves of the two PEMWEs. Compared with the sequentially sprayed layer, the PEMWE employing the conventional sprayed layer delivers a substantially lower current density of 3.2 A cm^−2^ at 2.0 V, corresponding to an approximately 31% performance enhancement enabled by the sequential spraying strategy. The sequentially sprayed anode layer also exhibits a 36.7% larger cyclic voltammetry (CV) area compared to the one-step sprayed anode layer (Supplementary Fig. 22). This result is consistent with the observed increase in activity, suggesting that the primary factor behind the improved performance is the enhanced exposure of active sites resulting from the reactivation of electrochemically inactive zones. Importantly, similar activity enhancements (25–35%) are also observed for sequentially sprayed anode layers prepared from three commercial IrO_2_ catalysts (Heraeus, YHKC, and Energy Chemical; Fig. 5c and Supplementary Fig. 23), demonstrating the general applicability of the sequential spraying strategy for boosting PEMWE anode performance. Furthermore, the performance enhancement achieved with the commercial titanium fiber felt is comparable to that observed in a visual electrolyzer based on an STP‑PTL (Fig. 4f), confirming the relevance of the visual observations and showing that the sequential spraying strategy is scalable to practical PTL structures.

Stability tests further demonstrated the practical viability of the sequentially sprayed catalyst layer (Fig. 5d). At a low iridium loading of 0.25 mg_Ir_ cm^−2^, the PEMWE equipped with the sequentially sprayed anode operates continuously for 3100 h at a current density of 1.0 A cm^−2^, followed by an additional 5250 h at 2.0 A cm^−2^. Over the entire durability test exceeding 8000 h (>11 months), the cell exhibits low voltage degradation rates of 3.4 and 12.4 µV h^−1^ at 1 and 2 A cm^−2^, respectively, demonstrating high long‑term durability. Top-view and cross-sectional SEM images (Supplementary Fig. 24) further confirmed that the catalyst layer structure remained intact after long-term operation, with no observable delamination or cracking, indicating sufficient mechanical robustness of the sequentially sprayed layer. In contrast, a PEMWE with a conventional one‑step sprayed anode under the same Ir loading suffers rapid performance decay and fails within 50 h at 2.0 A cm^−2^ (Supplementary Fig. 25).

Encouraged by the outstanding performance of the sequentially sprayed layer in the PEMWE single cell, we further evaluated its viability at the stack level. The fabrication of CCMs was scaled up to a large active area of 100 cm^2^ (Ir loading: 0.25 mg_Ir_ cm^−2^, Pt loading: 0.1 mg_Pt_ cm^−2^; Fig. 5e), while maintaining the same CCM preparation protocol used for the single cell. Notably, despite a 20-fold increase in membrane electrode area, the resulting CCMs exhibited highly uniform catalyst loadings across the entire active area (Supplementary Fig. 26), demonstrating the robustness and reliability of the sequential spraying process during scale-up.

The large-area CCMs were then integrated into a 2.1 kW PEMWE stack comprising six cells (Fig. 5f). Stack-level electrochemical performance was evaluated at 60 °C. As shown in Fig. 5g, the stack delivers high performance, achieving an average operating voltage of 1.75 V at a current density of 2.0 A cm^−2^. Moreover, the long-term operational stability of the sequentially sprayed catalyst layer was assessed under full-stack operating conditions. The stack operated stably for over 500 h at a current density of 2 A cm^−2^ (Fig. 5h), corresponding to a low average degradation rate of 60 μV h^−1^. Collectively, these results demonstrate the strong potential of the sequentially sprayed catalyst layer for scalable and durable PEMWE applications.

## Conclusions

This work has successfully identified that the limited in-plane electronic conductivity, originating from ionomer-induced disruption of the conductive network among IrO_2_ nanocatalysts, is the key factor restricting catalyst-layer utilization. Building on this insight, a sequential spray-coating strategy has been developed as a general approach to effectively activate in-plane inactive regions within the catalyst layer. Membrane electrode assemblies fabricated using this strategy exhibit three to four times enhancement in catalyst-layer electronic conductivity than those prepared by the conventional one-step spray method. Notably, this strategy surpasses the activity limits of traditional catalyst layers even at low iridium loadings while maintaining high stability, as validated at both the single-cell and stack levels. This work represents a significant advance in catalyst layer architecture, offering a scalable pathway toward high-performance, low-iridium electrolysis systems. Looking forward, this decoupling strategy can be readily extended to industrial coating methods such as doctor blading and roll-to-roll processing. In these processes, the catalyst layer is first deposited from an ionomer-free ink, and the ionomer is subsequently introduced via a low-viscosity impregnation step. This two-step strategy provides high flexibility for large-scale manufacturing.

## Methods

### Chemicals and reagents

Absolute ethanol (C_2_H_6_O), isopropanol ((CH_3_)_2_CHOH), and NaNO_3_ were purchased from Sinopharm Chemical Reagent Co., Ltd. Nafion^®^ perfluorinated resin solutions were obtained from Sigma-Aldrich (Dupont D520 and Dupont D521) and Shandong Dongyue Future Hydrogen Energy Material Co., Ltd (DY DH3105 and DY DH2205). IrCl_3_·H_2_O was supplied by Yurui (Shanghai) Chemical Co., Ltd. Commercial IrO_2_ catalysts were purchased from Heraeus Precious Metals Technology Co., Ltd. (Heraeus IrO_2_), Energy Chemical Co., Ltd. (Energy IrO_2_), and Ningbo Zhongke Kecheng New Energy Technology Co., Ltd. (YHKC IrO_2_). Pt/C catalyst (40 wt% Pt) was obtained from Johnson Matthey. Highly purified water (>18 MΩ cm resistivity) was produced using a PALL PURELAB Plus system.

### Synthesis of IrO_2_ catalyst

The in-house synthesized IrO_2_ catalyst was synthesized by an Adams melting method^37^. Briefly, 0.37 g of IrCl_3_·H_2_O and 1.1 g of NaNO_3_ was dissolved in 20 mL of isopropanol to form a homogeneous solution. The mixture was dried at 80 °C for 5 h to remove the solvent, followed by calcination at 350 °C for 1 h. The resulting powder was immersed in 0.5 M H_2_SO_4_ to remove residual NaCl species, then washed with deionized water and ethanol. Finally, the product was dried at 80 °C to obtain the IrO_2_ catalyst.

### Characterizations

XRD patterns were conducted by a Bruker D8 Advance diffractometer with Cu Kα radiation (*λ* = 1.5418 Å). Low-magnification TEM images were obtained on a Philips-FEI Tecnai G2S-Twin microscope equipped with a field emission gun operating at 200 kV. SEM images were obtained with a field emission SEM (JEOL 7800F), and Elemental mapping analysis was carried out on the connected system. DLS measurements were conducted on a Malvern Zetasizer Nano-ZS90 instrument (detection range: 3−3,000 nm). The HFR of the PEMWE cells was measured using the Gamry instrument with a 30 A booster.

### Catalyst-coated membrane (CCM) fabrication

Prior to CCM fabrication, Nafion 115 was sequentially treated with 3 wt % H_2_O_2_, deionized water, and 0.5 M H_2_SO_4_ at 80 °C for 1 h each, followed by thorough rinsing with deionized water.

#### One-spray strategy (conventional method)

For conventional CCM fabrication, an anode catalyst ink was prepared by dispersing IrO_2_ catalyst in a mixed solution of isopropyl alcohol and deionized water (2:1, w/w). A Nafion ionomer solution was then added to the dispersion, with the ionomer content fixed at 15 wt% relative to the catalyst unless otherwise specified. The mixture was sonicated for 1.5 h to obtain a homogeneous ink, which was subsequently sprayed onto the PEM to form the ACL. No conductive or stabilizing supports were used in any of the catalyst layers studied in this work.

#### Sequential spray strategy

In this approach, an ionomer‑free catalyst ink was first prepared by dispersing IrO_2_ catalyst in a mixed solution of isopropyl alcohol and deionized water (1.5:1, w/w), followed by sonication to achieve a uniform dispersion. Separately, a Nafion dilution was prepared by mixing a 5 wt% Nafion^®^ perfluorinated resin solution in isopropyl alcohol with a volume ratio of 1:40. The catalyst ink was deposited onto the PEM via ultrasonic spraying. Subsequently, the Nafion dilution was sprayed onto the pre‑formed catalyst layer to obtain an ionomer content of 15 wt% relative to the catalyst. To prepare a high-loading catalyst layer (>0.25 mg_Ir_ cm^−2^), the strategy can be readily extended by repeating the “catalyst spray/ionomer spray” cycle two or more times. Specifically, thin IrO_2_ sublayers are sequentially deposited and infiltrated with ionomer in a stepwise manner to gradually build up the total catalyst loading.

For the cathode, a commercial Johnson Matthey Pt/C catalyst (40 wt% Pt) was used. The cathode catalyst layer was fabricated using the one‑spray strategy and applied to the opposite side of the PEM. The resulting CCM, comprising an IrO_2_ ACL and a Pt/C cathode catalyst layer, was then hot‑pressed at 130 °C under 10 MPa for 10 min. The precious metal mass loading was determined by X-ray fluorescence spectrometer analysis.

### Conductivity measurement of the catalytic layer

The in-plane electrical conductivity of the anode catalytic layer was measured via a standard four-probe method. To prevent mechanical damage to the catalytic layer during measurement, four metal electrodes with uniform size and spacing were fabricated directly on the membrane electrode by an electron-beam evaporation system (AMOD, Canada) in combination with a mask plate method. The mask plate contained four circular apertures with a diameter of 2 mm and a center-to-center spacing of 0.2 mm. Electrode deposition was carried out in two sequential steps: a 20 nm titanium adhesion layer was first deposited, followed by a 60 nm silver conductive layer. A bench-top power supply integrated with a four-point probe system (Keithley Instruments, 2440-C 5A Source Meter^®^) was employed to apply a small probing current, while the corresponding voltage was recorded in the range of 1–150 mV.

The bulk resistance *R*_s_ (Ω) of the anode catalytic layer was calculated based on the classical four-probe theory according to Eq. (1)^38^, where *I* is the applied current and *V* is the average measured voltage:

1 $${R}_{s}=\frac{\pi }{{{\mathrm{ln}}}(2)}\frac{V}{I}=4.5324\times \frac{V}{I}$$

Bulk resistivity *ρ* (Ω • m) of the catalytic layer was then determined using Eq. (2):

2 $$\rho={R}_{s}\times {{{\rm{d}}}}$$

Where d represents the thickness of the catalytic layer.

Electrical conductivity *σ* (S m^−1^) of the anode catalytic layer was determined using Eq. (3):

3 $$\sigma=1/\rho$$

### Electrochemical measurement of PEMWE

#### Single-cell PEMWE measurement

The electrochemical performance of the PEMWE single cell was evaluated using a commercial test integration unit (Hefei Conservation of Momentum Green Energy Co., Ltd.). Nafion 115 was employed to fabricate the CCM with an active area of 5 cm^2^. A 0.26 mm-thick titanium felt coated with a 2 μm platinum layer and a 0.2 mm-thick carbon paper were used as the anode and cathode PTLs, respectively. The PEMWE is assembled by tightening the bolts to 5 N m, unless otherwise specified.

Prior to testing, the assembled PEMWE cell was continuously flushed with ultrapure water (measured pH 6.917 ± 0.05, determined from three parallel measurements, and reported as pH 7 throughout this work) at 80 °C for at least 5 h using a circulating peristaltic pump to ensure complete system conditioning. CCM activation was subsequently carried out under galvanostatic operation at current densities of 0.1 and 0.5 A cm^−2^, each maintained for 1 h. Polarization curves were recorded by linear sweep voltammetry at a scan rate of 20 mV s^−1^ over a potential range of 1.4–2.1 V using a Gamry electrochemical workstation equipped with a 30 A current booster. Long-term durability was assessed by chronopotentiometry using a NEWARE battery testing system (CT-4008-5V100A). The ohmic resistance of the PEMWE cell corresponds to the HFR, which is obtained via EIS. All electrochemical parameters were controlled by the Gamry instrument equipped with a 30 A booster. The frequency range was set from 0.05 to 20,000 Hz, the alternating current was 0.091 A rms, the direct current was 1 A, and the catalytic area was 5 cm^2^.

#### Six-cell PEMWE stack measurement

A six-cell PEMWE stack was assembled using a DM6525 PEM. The CCMs were prepared using in-house synthesized IrO_2_ via the sequential spray strategy. Each single-cell CCMs had an active area of 100 cm^2^ (10 cm × 10 cm), with iridium and platinum loadings of 0.25 and 0.1 mg cm^−2^, respectively. For the PTLs, a 200 μm-thick titanium PTL plated with platinum was employed on the anode side, while a 180 μm-thick carbon paper (Toray) was used on the cathode side. The stack was assembled by sequentially stacking the layers from anode to cathode.

The test is conducted at a constant temperature of 60 °C without external auxiliary heating. After the initial activation, the polarization curve of the entire stack is tested. After 10 min of standing +10 min of 1 A constant current +10 s of constant current gradually increasing, and sampling points are reduced as the current density increases to prevent overheating, the test reaches 4 A cutoff. This step is repeated for repeated testing until the polarization performance reaches the optimal or becomes stable. The stability test is 100 h of 2 A constant current +5 h of standing, with a cumulative test duration of 550 h.

### In-situ visualization measurement of gas bubble evolution

The bubble evolution was investigated using a self‑built visualization system, which comprised a transparent, reaction-visible PEMWE (Hefei Conservation of Momentum Green Energy Co., Ltd.), a high-speed camera (Phantom VEO711), a long-working-distance microscope (Infinity Model K2 DistaMaxTM), and a NEWARE battery testing system (CT-4008-5V100A, Shenzhen, China). Image acquisition was performed at a resolution of 512 × 512 pixels, a sampling rate of 10,000 frames per second, an exposure time of 71 μs, and an exposure index of 32,000, which was used to capture the bubble generation and detachment processes on the electrode under operating conditions. All components are mounted on an anti-vibration optical table to ensure stability and accuracy. In addition, an adjustable-intensity cold light source provided by a gooseneck probe ensures high-quality videos and images. To facilitate quantitative analysis of bubble dynamics, ultrapure water was circulated through the electrolyzer at a controlled constant flow rate of 20 mL min^−1^.

### Computation methods

To elucidate the molecular-scale behavior of the ionomer-IrO_2_ system, molecular dynamics simulations were performed using the DFTB+ package with the integrated GFN2-xTB method^39,40^. The simulation cell had dimensions of 20 × 14 × 36 Å^3^ and contained two (IrO_2_)_3_ clusters^41^, one Nafion polymer chain^42^, 80 water molecules, and 10 isopropanol molecules, representing a model catalyst ink environment.

An initial solvent pre-equilibration was carried out for 4 ps, during which the IrO_2_ clusters and the ionomer polymer chain were held fixed while the solvent molecules were allowed to relax. Subsequently, all constraints were released, and a 7 ps production simulation was performed. Both the pre-equilibration and production stages were conducted in the canonical (NVT) ensemble. Temperature control was achieved using a Nosé-Hoover chain thermostat, maintaining the system at 300 K. A time step of 1 fs was employed throughout all simulations.

## Supplementary information

Supplementary Information

Description of Additional Supplementary Files

Supplementary Data 1

Supplementary Video 1

Supplementary Video 2

Supplementary Video 3

Supplementary Video 4

Supplementary Video 5

Supplementary Video 6

Supplementary Video 7

Transparent Peer Review file

## Source data

Source data

## Data Availability

The data that support the findings of this study are available within the article and its Supplementary Information. All other relevant data supporting the findings of this study are available from the corresponding authors upon request. Source data are provided with this paper.
