# Supplementary material for: Activating in-plane dead zones of anode catalyst layers in proton exchange membrane water electrolyzers
Source: Nat Commun. 2026 Jul 1;17:8211. doi: 10.1038/s41467-026-75223-1 (PMC13462438; doi:10.1038/s41467-026-75223-1)
Supplement: Supplementary file 1 [file 41467_2026_75223_MOESM11_ESM.pdf]

nature portfolio

Peer Review File

Activating In-Plane Dead Zones of Anode Catalyst Layers in Proton Exchange Membrane Water Electrolyzers

Corresponding Author: Professor Xiaoxin Zou

This file contains all reviewer reports in order by version, followed by all author rebuttals in order by version.

Version 0:

Reviewer comments:

Reviewer #1

(Remarks to the Author)
This work reports a sequential spray-coating strategy to activate the electrochemically inactive "dead zones" in the anode catalyst layer of PEM water electrolyzers. By decoupling the construction of the conductive IrO_2 skeleton from the ionomer infiltration, the authors successfully improved the in-plane electronic conductivity and achieved a high current density of 4.2 A cm^{-2} at 2.0 V with a low Ir loading of 0.25 mg cm^{-2}. Through in situ visualization and quantitative conductivity analysis, the paper provides solid evidence that the ionomer wrapping effect is the main cause of the dead zones. However, the stability of the proposed structure under dynamic conditions and the actual conductivity in a wet environment need further verification. In addition, the practical scalability of the process requires more discussion. Therefore, a Major Revision is recommended before it can be considered for publication on Nature Communications. Detailed comments are listed below:
(1) The in-plane electronic conductivity measurements in Figure 3 and Figure 4 were likely performed under dry conditions. However, in a real electrolyzer, the catalyst layer is fully hydrated, and the ionomer swells significantly. The authors should discuss or measure how hydration affects the electronic connectivity of the "skeleton" formed in the sequential spray method compared to the conventional mixed ink.
(2) The sequential spray strategy relies on the infiltration of the dilute ionomer solution into the pre-sprayed catalyst layer. While this works for low loadings (0.25 mg cm^{-2}), it is important to clarify the limit of this penetration. If the catalyst layer becomes thicker (e.g., higher loading or lower density support), can the ionomer still reach the membrane interface effectively without blocking the pores?
(3) Regarding mechanical stability, the first step involves spraying pure IrO_2 nanoparticles without any binder. The authors should provide evidence (such as a peel test or cross-sectional SEM after long-term operation) to prove that the catalyst layer has sufficient adhesion to the membrane and does not delaminate under gas evolution pressure.
(4) In Figure 2f, the visualization shows that at 0% ionomer content, bubbles are generated across the entire open area. The authors should explicitly explain why this condition (0% ionomer) is not ideal for overall cell performance, emphasizing the trade-off between electronic conductivity (activating dead zones) and proton conductivity (essential for reaction kinetics).
(5) The paper claims to activate the "dead zones" in the center of the PTL pores. While this increases the active area, mass transport in these center regions might be poor at high current densities (>2 A cm^{-2}). The authors should discuss whether the bubbles generated in the center can be effectively removed or if they cause gas blinding at high rates.
(6) The solvent usage in the sequential spray method seems very high (1:40 dilution for the ionomer). From a practical and industrial perspective, the authors should comment on the scalability and environmental impact of using such a large amount of solvent compared to the conventional one-pot method.
(7) For the visualization setup (Figure 2), the clamping pressure is a critical parameter affecting contact resistance and bubble dynamics. The authors should clarify if the pressure used in the visualization cell is comparable to the standard cell testing (Figure 5) and how pressure might affect the "dead zone" distribution.
(8) There is a minor inconsistency in the terminology regarding the "dead zone" threshold. In the text, the threshold is stated as ~63 S m^{-1}. The authors should clarify if this value is a universal constant for IrO_2-based layers or if it depends on the specific geometry (pore size) of the PTL used in this study.

Reviewer #2

(Remarks to the Author)
Manuscript NCOMMS-26-014197-T presents a well-structured study on the influence of the ionomer addition sequence during the fabrication of MEAs for PEM water electrolysis. Specifically, it compares the traditional one-step approach where

the ionomer is incorporated directly into the catalyst ink, with a sequential method in which the catalyst layer is deposited first without ionomer and the ionomer is added only afterward. The results presented in this work show that the sequential strategy yields significantly higher performance, regardless of catalyst properties or I/C ratio, which constitutes a relevant contribution to MEA optimization. The study provides compelling indications that adding the ionomer already in the catalyst ink affects catalyst distribution and induces in-plane electronic conductivity limitations, giving rise to inactive regions ("dead zones"), while sequential deposition improves electronic percolation which according to the authors, results in higher electrocatalytic activity.

One of the most striking observations in the manuscript is the long-term stability of the ionomer-free IrO_2 inks, which reportedly remain stable for several months. This behavior is unexpected, given the well-established role of ionomer in stabilizing catalyst dispersions, and therefore requires further explanation. Additionally, clarification is needed regarding whether conductive or stabilizing supports, such as ATO, were used in the ink formulations. These materials are commonly employed to enhance both dispersion stability and in-plane conductivity, especially at low Ir loadings (around 0.5 mg/cm2 or lower). This point is also relevant because several of the observed effects such as the extent and mitigation of inactive regions, may differ substantially when using supported catalysts like Ir/ATO or similar. A discussion on whether the reported behavior persists in the presence of partially conductive supports would significantly strengthen the manuscript.

The study employs an in situ visualization technique to observe bubble evolution within the PTL and at the catalyst layer interface. While experimentally elegant and informative, the manuscript should more clearly define what phenomena are actually captured by this system. In some parts of the text, see for instance the caption to Figure 2 and lines 100-101 stating that "the electrochemical reaction in operating PEMWE was investigated using a self-built visualization system", the authors suggest that electrochemical reactions are directly visualized during operation; however, the images and videos provided appear to show only bubble formation and growth, not the reaction at catalytic active sites. It is well understood that most oxygen generated at catalytic sites initially dissolves into the electrolyte before forming bubbles, and thus these observations should not be interpreted as direct visualization of the reaction itself. Clarification is also needed regarding the significance of highlighted regions in the images (such as colored spots or contours) and how these relate to bubble dynamics.

The results presented here indicate that ionomer incorporation during deposition can lead to electronically insulated regions that reduce catalyst utilization. However, the claim that the sequential method "activates previously inactive regions" is not yet fully supported by the presented evidence, since the catalytic activity (or lack of thereof) in any region has not been established for any system. The conductivity measurements indicate improvements, but more direct or quantitative evidence of increased catalyst utilization would be necessary, or alternatively the claim should be reconsidered accordingly.

Also, it is important to consider the representativeness of the actual setup used for the visualization system. The highly macroporous PTLs used are ideal for enabling optical access, but both their morphology and porous structure differ substantially from the PTLs typically used in commercial and industrial PEMWE systems. The manuscript should therefore discuss the extent to which the visual observations can be extrapolated to conventional PTLs, and the limitations this may impose on the generality of the findings.

Several experimental aspects also require greater clarity. It is recommended that operating conditions (such as voltage) be stated explicitly in the relevant figures (e.g., Figures 5a and 5c). The degradation behavior shown for the one-step sample in Figure 5d appears unusually rapid for IrO_x-based catalysts; clarification regarding the reproducibility of this result and whether it represents typical behavior would be valuable. Furthermore, the comparison of ionomer distribution between the one-step and sequential samples raises questions: the absence of a fluorine-rich overlayer in the sequential case is counterintuitive, and a more detailed explanation of ionomer penetration and distribution within the catalyst layer would improve the mechanistic narrative.

Finally, since the sequential deposition strategy is presented as potentially scalable, it would be beneficial for the manuscript to discuss its compatibility with industrially relevant coating processes such as doctor blading or roll-to-roll manufacturing in which ink viscosity, wettability, and uniformity are tightly coupled to ionomer content. Despite these points requiring clarification, the manuscript offers a meaningful conceptual advance and a promising experimental approach, and addressing the issues outlined above would significantly strengthen its impact.

Reviewer #3

(Remarks to the Author)
I co-reviewed this manuscript with one of the reviewers who provided the listed reports. This is part of the Nature Communications initiative to facilitate training in peer review and to provide appropriate recognition for Early Career Researchers who co-review manuscripts.

Version 1:

Reviewer comments:

Reviewer #1

(Remarks to the Author)
The revision is sufficient for acceptance.

Reviewer #2

(Remarks to the Author)
The authors have addressed all of our remarks satisfactorily, and have properly included all modifications in the manuscript. Therefore, the manuscript can be published as is.

Reviewer #3

(Remarks to the Author)
I co-reviewed this manuscript with one of the reviewers who provided the listed reports. This is part of the Nature Communications initiative to facilitate training in peer review and to provide appropriate recognition for Early Career Researchers who co-review manuscripts.

Open Access This Peer Review File is licensed under a Creative Commons Attribution 4.0 International License, which permits use, sharing, adaptation, distribution and reproduction in any medium or format, as long as you give appropriate credit to the original author(s) and the source, provide a link to the Creative Commons license, and indicate if changes were made.
In cases where reviewers are anonymous, credit should be given to 'Anonymous Referee' and the source.
The images or other third party material in this Peer Review File are included in the article's Creative Commons license, unless indicated otherwise in a credit line to the material. If material is not included in the article's Creative Commons license and your intended use is not permitted by statutory regulation or exceeds the permitted use, you will need to obtain permission directly from the copyright holder.
To view a copy of this license, visit https://creativecommons.org/licenses/by/4.0/

REVIEWER COMMENTS

Reviewer #1 (Remarks to the Author):

This work reports a sequential spray-coating strategy to activate the electrochemically inactive "dead zones" in the anode catalyst layer of PEM water electrolyzers. By decoupling the construction of the conductive IrO_2 skeleton from the ionomer infiltration, the authors successfully improved the in-plane electronic conductivity and achieved a high current density of 4.2 A cm^{-2} at 2.0 V with a low Ir loading of 0.25 mg cm^{-2}. Through *in situ* visualization and quantitative conductivity analysis, the paper provides solid evidence that the ionomer wrapping effect is the main cause of the dead zones. However, the stability of the proposed structure under dynamic conditions and the actual conductivity in a wet environment need further verification. In addition, the practical scalability of the process requires more discussion. Therefore, a Major Revision is recommended before it can be considered for publication on Nature Communications. Detailed comments are listed below:

Response: We thank the reviewer for the positive evaluation of our work and for recognizing the significance of the sequential spray-coating strategy in activating the electrochemically inactive "dead zones" within the PEMWE anode catalyst layer. We also appreciate the reviewer's constructive comments regarding the stability, conductivity under hydrated conditions, and practical scalability of the proposed structure. We have carefully addressed these concerns in the revised manuscript. Detailed point-by-point responses are provided below.

(1) The in-plane electronic conductivity measurements in Figure 3 and Figure 4 were likely performed under dry conditions. However, in a real electrolyzer, the catalyst layer is fully hydrated, and the ionomer swells significantly. The authors should discuss or measure how hydration affects the electronic connectivity of the "skeleton" formed in the sequential spray method compared to the conventional mixed ink.

Response 1: We thank the reviewer for this insightful comment. We agree that the in-plane electronic conductivity measurements shown in Figure 3 and Figure 4 were performed under dry conditions. These measurements were intentionally conducted under dry conditions to minimize environmental interference and enable a direct comparison of the intrinsic electronic connectivity within the catalyst layers. The results clearly demonstrate that the sequential spray method improves the electronic conductivity of catalyst layer by constructing a more effective conductive skeleton.

To further address the reviewer's concern regarding conductivity under realistic operating conditions, we additionally analyzed the high-frequency resistance (HFR) derived from electrochemical impedance spectroscopy (EIS) during PEMWE operation (**Figure R1 or Supplementary Fig. 20**). The HFR reflects the total ohmic resistance of the cell, including contributions from the membrane, catalyst layers, porous transport layers, bipolar plates, and their interfaces. Importantly, all cell components were

identical in our comparison except for the anode catalyst layer fabrication method. The PEMWE employing the sequentially sprayed anode exhibited an HFR of 110 mΩ cm^2, which is significantly lower than the 131 mΩ cm^2 measured for the conventional one-step sprayed anode. Since the anode catalyst layer structure is the only variable, this lower HFR directly confirms that the enhanced electronic connectivity established by the sequential spray strategy is maintained under fully hydrated operating conditions.

Therefore, the dry-state in-plane conductivity measurements together with the *in situ* wet-state HFR results consistently demonstrate the superior electronic transport properties enabled by the sequential spray method.

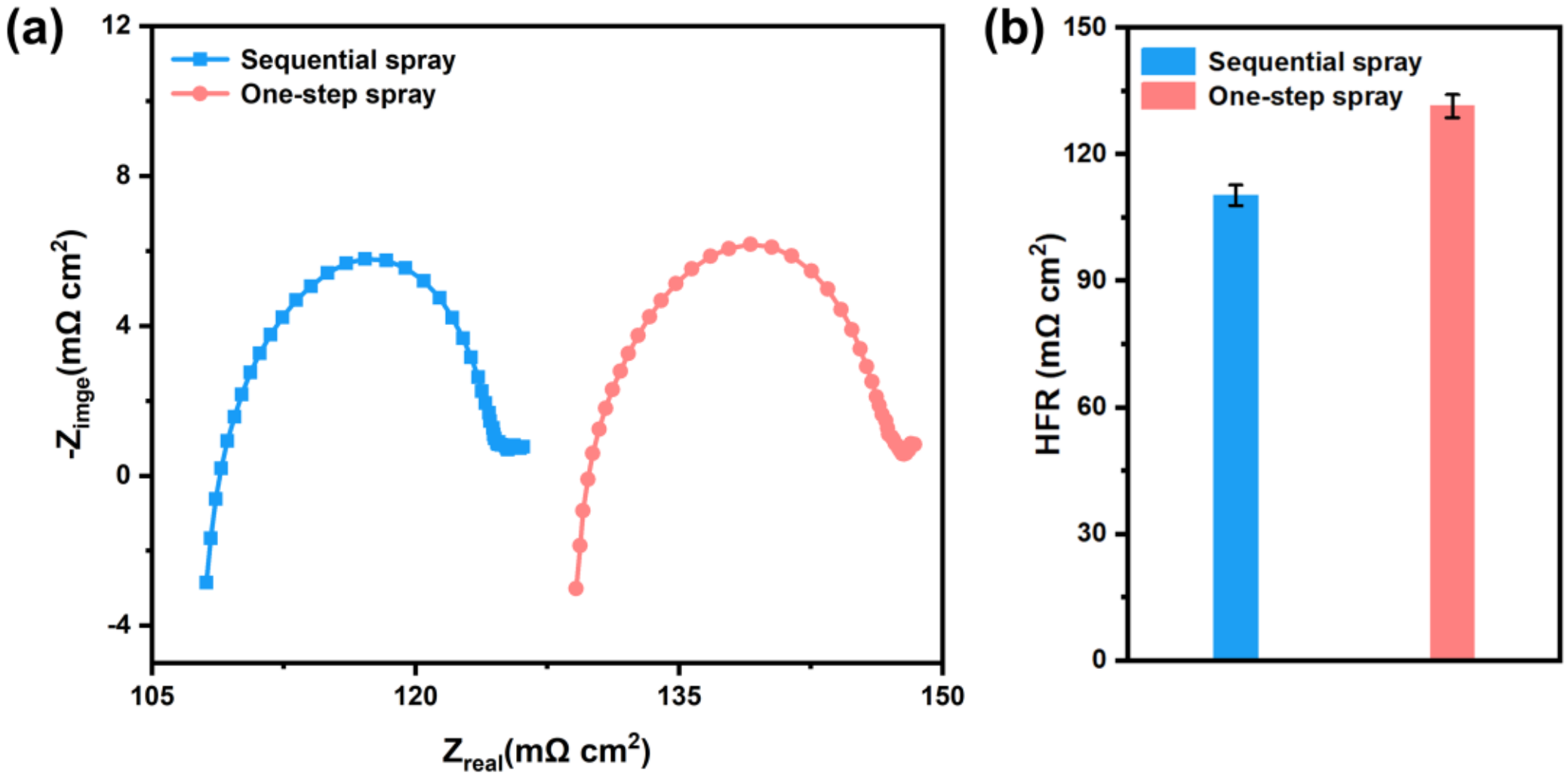

Figure R1 (a) Electrochemical impedance spectroscopy (EIS) spectra and (b) corresponding high-frequency resistance (HFR) values of PEMWE cells employing sequentially sprayed and one-step sprayed anode catalyst layers.

(2) The sequential spray strategy relies on the infiltration of the dilute ionomer solution into the pre-sprayed catalyst layer. While this works for low loadings (0.25 mg cm^{-2}), it is important to clarify the limit of this penetration. If the catalyst layer becomes thicker (e.g., higher loading or lower density support), can the ionomer still reach the membrane interface effectively without blocking the pores?

Response 2: We thank the reviewer for raising this important point. In our method, an IrO_2 catalyst layer with a loading of 0.25 mg_{Ir} cm^{-2} is first sprayed, followed by the deposition of ionomer solution (15 wt%) onto the pre-formed catalyst layer. At this loading, the catalyst layer is thin to allow the ionomer to uniformly infiltrate throughout the entire thickness.

For higher catalyst loadings or thicker catalyst layers, the strategy can be readily extended by repeating the “catalyst spray/ionomer spray” cycle two or more times. Specifically, thin IrO_2 sublayers are sequentially deposited and infiltrated with ionomer in a stepwise manner to gradually build up the total catalyst loading. Because each individual sublayer remains thin, uniform ionomer penetration can still be maintained, ensuring effective ionomer distribution throughout the catalyst layer and efficient contact with the membrane interface.

Therefore, the sequential spray strategy is not fundamentally limited by catalyst layer thickness and, in principle, is compatible with higher loading. We have clarified this point in the revised manuscript (**Page 17**) to avoid misunderstanding.

(3) Regarding mechanical stability, the first step involves spraying pure IrO_2 nanoparticles without any binder. The authors should provide evidence (such as a peel test or cross-sectional SEM after long-term operation) to prove that the catalyst layer has sufficient adhesion to the membrane and does not delaminate under gas evolution pressure.

Response 3: To directly evaluate adhesion and structural integrity, we characterized the membrane electrode after long-term operation (120 hours at 2 A cm^{-2}). Top-view and side view SEM images (**Figure R2 or Supplementary Fig. 24**) confirm that the catalyst layer structure remains intact, with no signs of delamination or cracking. This demonstrates that the sequentially sprayed layer maintains sufficient mechanical robustness (**Page 15**).

The key to this stability lies in the subsequent ionomer infiltration step. Although the first step deposits pure IrO_2 nanoparticles without a binder, the ionomer solution sprayed in the second step effectively permeates the pre-formed particle network. Upon drying, the ionomer acts as a binder, physically holding the IrO_2 particles together and anchoring the entire layer to the membrane.

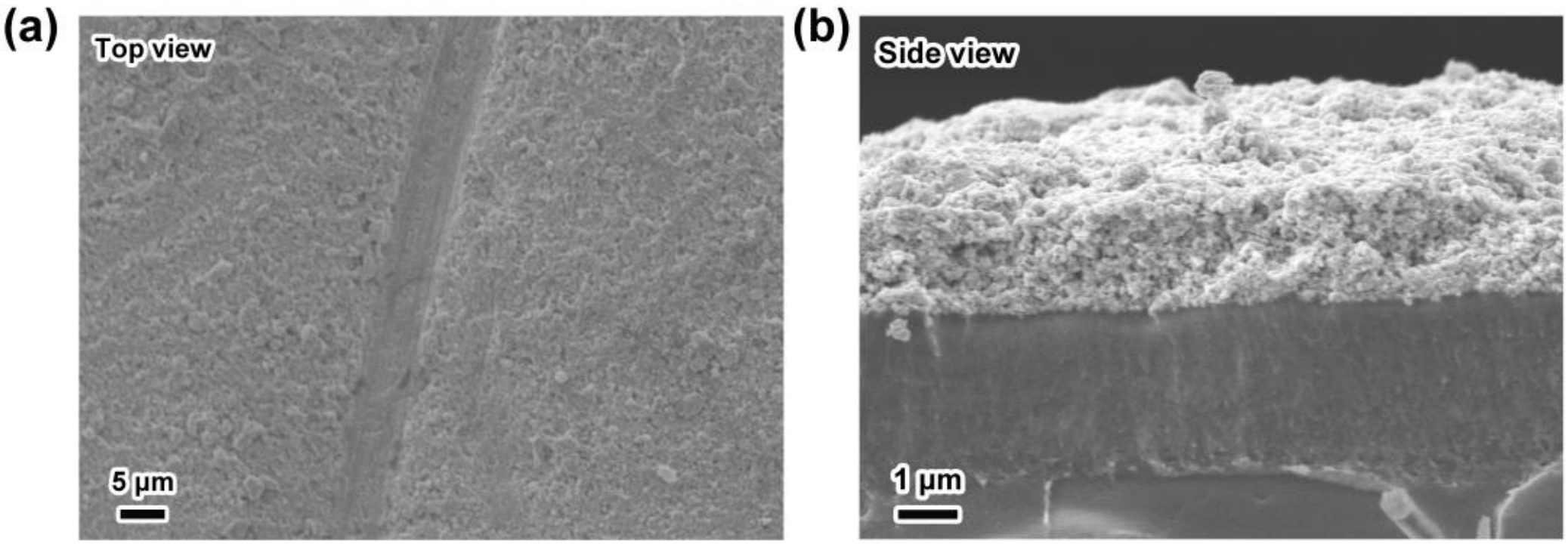

Figure R2 (a) Top-view and (b) side view SEM images of sequentially sprayed layer after long-term operation (120 hours at 2 A cm^{-2}).

(4) In Figure 2f, the visualization shows that at 0% ionomer content, bubbles are generated across the entire open area. The authors should explicitly explain why this condition (0% ionomer) is not ideal for overall cell performance, emphasizing the trade-off between electronic conductivity (activating dead zones) and proton conductivity (essential for reaction kinetics).

Response 4: We thank the reviewer for this insightful suggestion. We indeed tested the PEMWE performance of the catalyst layer with 0% ionomer content. This condition initially delivers a high activity of 2 A cm^{-2} at 1.74 V (**Figure R3a or Supplementary Fig. 15**), confirming that the absence of ionomer eliminates the “dead zones” and

maximizes electronic connectivity. However, the cell degrades severely within 30 hours of operation (**Figure R3b or Supplementary Fig. 16**).

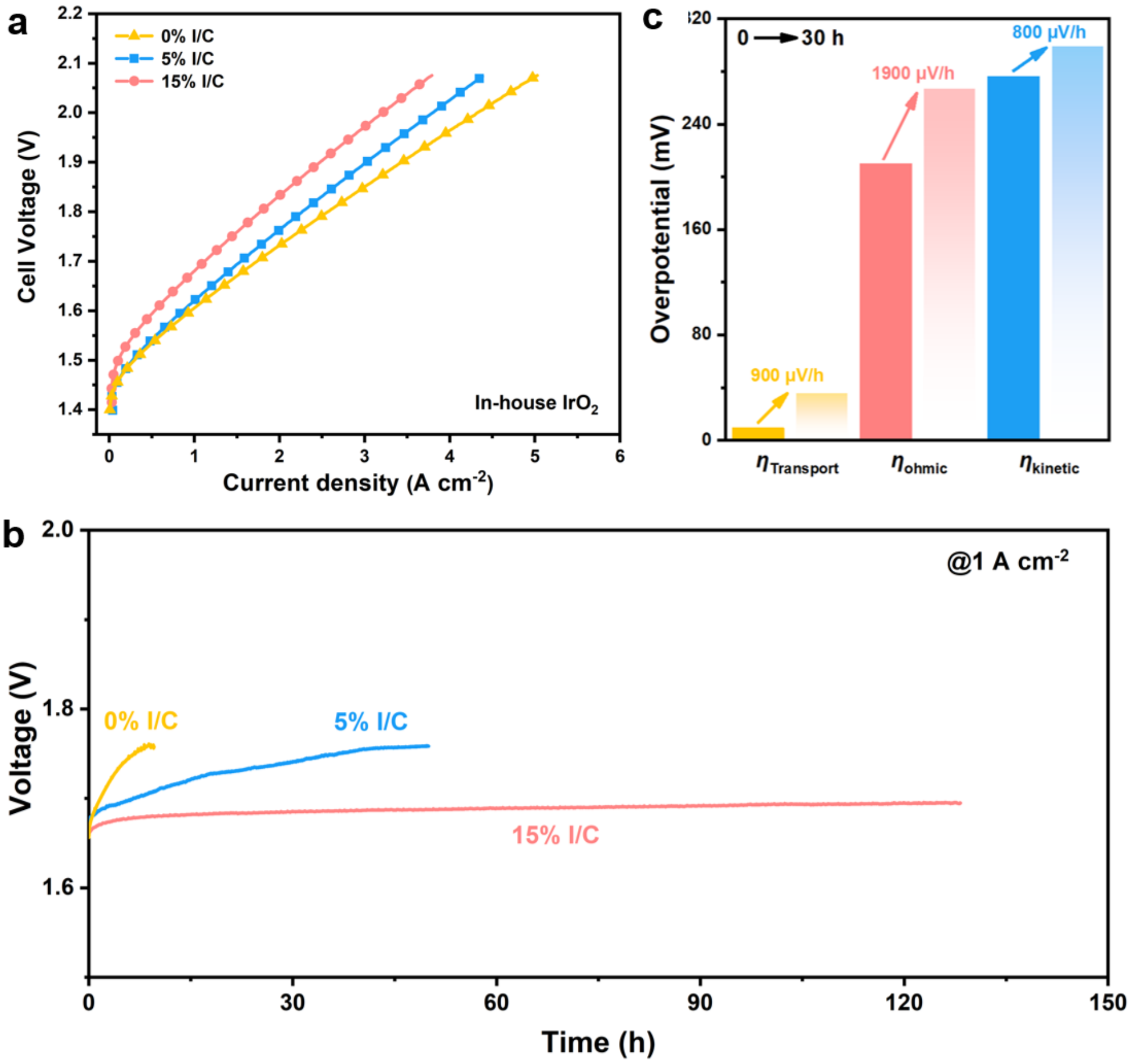

Figure R3 (a) Chronopotentiometry curve of PEMWE using 0% ionomer anode. (b) Voltage losses before and after long-term stability test on the 0% ionomer anode cell.

To investigate the degradation mechanism, we performed voltage loss analysis after prolonged operation on the 0% ionomer anode cell (**Figure R3c**). After 30 h, the cell exhibits rapid degradation in all three overpotentials: 800 μV h^{-1} for kinetic, 1900 μV h^{-1} for ohmic, and 900 μV h^{-1} for transport losses. This pronounced instability arises from severe agglomeration and detachment of IrO_2 nanoparticles under PEMWE operating conditions. Without ionomer acting as a binder and proton conductor, the IrO_2 nano-aggregates undergo maturation and coalescence, which dramatically reduces electrochemically active site accessibility, creates larger electrically isolated dead zones, and obstructs gas/liquid transport pathways.

This result illustrates the critical trade-off: while eliminating ionomer maximizes initial electronic conductivity, the complete absence of proton conduction pathways and the mechanical instability of a binder-free particle network render the electrode unsustainable. Our sequential spray strategy resolves this trade-off by first building a percolated IrO_2 skeleton for high electronic conductivity, and then infiltrating ionomer to establish proton transport and provide structural integrity, thereby achieving both high initial performance and durable operation.

(5) The paper claims to activate the "dead zones" in the center of the PTL pores. While this increases the active area, mass transport in these center regions might be poor at high current densities (>2 A cm^{-2}). The authors should discuss whether the bubbles generated in the center can be effectively removed or if they cause gas blinding at high rates.

Response 5: To address the concern, we performed two complementary sets of experiments. First, we decoupled the total cell voltage into activation, ohmic, and mass-transport overpotentials over a wide current density range of 1–5 A cm^{-2}. The mass-transport overpotential consistently accounts for only 4–6 % of the total voltage loss (**Figure R4**), which is a strong indicator of highly efficient mass transport even at very high current densities. Second, we directly visualized the bubble dynamics at 2 A cm^{-2} using a self-built visualization system (**Supplementary Video 7**). The results confirm that bubbles generated in the central regions of the PTL pores are effectively removed.

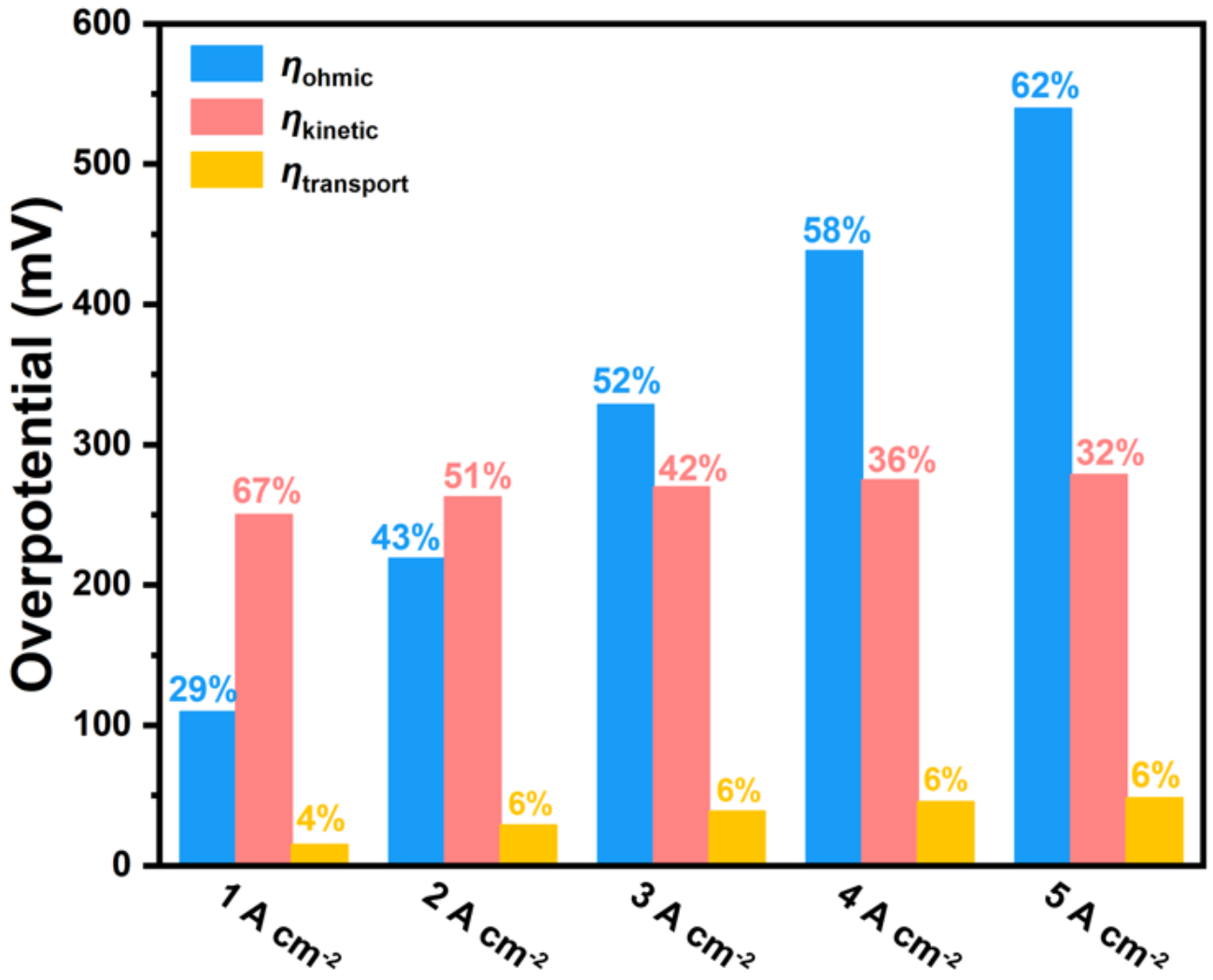

Figure R4 Overpotential analysis detailing the ohmic overpotential (η_{ohm}), kinetic overpotential (η_{kin}), and transport overpotential (η_{trans}) contributions for PEMWEs using sequentially sprayed layer with different current densities (80 °C, Nafion 115).

(6) The solvent usage in the sequential spray method seems very high (1:40 dilution for the ionomer). From a practical and industrial perspective, the authors should comment on the scalability and environmental impact of using such a large amount of solvent compared to the conventional one-pot method.

Response 6: We appreciate the Reviewer's practical perspective on scalability and environmental impact. It is important to clarify that the high dilution factor (1:40) used in this study is not unique to the sequential spray method. For laboratory-scale fabrication of small-area membrane electrode *via* ultrasonic spraying, the conventional one-pot method also employs comparably dilute inks, resulting in similar solvent usage. At the industrial scale, both approaches can be adapted to roll-to-roll coating processes with significantly higher solids content, thereby drastically reducing solvent

consumption.

The essential novelty of the sequential spray method lies not in the specific ink concentrations or spraying parameters, but in its design principle: it decouples the deposition of a pristine conductive catalyst layer from the ionomer, preserving continuous electron transport pathways. Therefore, this concept can guide the development of more suitable, industrially viable ink formulations for large-scale manufacturing. We have added a comment on this point in the revised manuscript (**Page 16**).

(7) For the visualization setup (Figure 2), the clamping pressure is a critical parameter affecting contact resistance and bubble dynamics. The authors should clarify if the pressure used in the visualization cell is comparable to the standard cell testing (Figure 5) and how pressure might affect the "dead zone" distribution.

Response 7: We thank the Reviewer for raising this important point. We confirm that the pressure applied in the visualization cell (bolt torque: 5 N·m; corresponding clamping pressure: 3.5 MPa) is identical to that used in standard cell testing, ensuring full comparability between the two setups. We have added a clarification in the revised manuscript (**Page 18**).

Regarding the effect of pressure on the "dead zone" distribution, we performed additional experiments at bolt torques of 1 N·m, 2 N·m, 5 N·m and 7 N·m. Variations in clamping pressure (**Supplementary Video 5**) all yield the same pattern that electrochemical reaction remains confined to the boundary region of the porous transport layer and the anode catalyst layer. These results demonstrate that the formation of in-plane dead zones is a universal phenomenon, independent of clamping pressure.

(8) There is a minor inconsistency in the terminology regarding the "dead zone" threshold. In the text, the threshold is stated as ~63 S m^{-1}. The authors should clarify if this value is a universal constant for IrO_2-based layers or if it depends on the specific geometry (pore size) of the PTL used in this study.

Response 8: We thank the Reviewer for this careful observation. The threshold of ~63 S m^{-1} is not a universal constant for all IrO_2-based layers. This value was derived from the specific PTL geometry employed in this study, and a different PTL geometry would likely shift this threshold.

Importantly, this does not affect our fundamental understanding of the origin of the “dead zones” or the validity of the proposed activation strategy. The underlying mechanism that ionomer disrupts the conductive network of IrO_2 nanocatalysts and creates in-plane dead zones is general, and the strategy of decoupling the ionomer from the catalyst layer to activate these zones remains broadly applicable. Only the specific numerical threshold needs to be re-evaluated when the PTL structure changes. We have revised the manuscript to explicitly clarify this point (**Page 8**).

Reviewer #2 (Remarks to the Author):

Manuscript NCOMMS-26-014197-T presents a well-structured study on the influence of the ionomer addition sequence during the fabrication of MEAs for PEM water electrolysis. Specifically, it compares the traditional one-step approach where the ionomer is incorporated directly into the catalyst ink, with a sequential method in which the catalyst layer is deposited first without ionomer and the ionomer is added only afterward. The results presented in this work show that the sequential strategy yields significantly higher performance, regardless of catalyst properties or I/C ratio, which constitutes a relevant contribution to MEA optimization. The study provides compelling indications that adding the ionomer already in the catalyst ink affects catalyst distribution and induces in-plane electronic conductivity limitations, giving rise to inactive regions ("dead zones"), while sequential deposition improves electronic percolation which according to the authors, results in higher electrocatalytic activity.

Response: We sincerely thank the Reviewer for the highly positive and insightful assessment of our work. We are truly encouraged that the Reviewer recognizes the well-structured study design, the systematic comparison between the conventional one-step and sequential ionomer addition methods, and the compelling evidence for the "dead zone" activation mechanism.

(1) One of the most striking observations in the manuscript is the long-term stability of the ionomer-free IrO_2 inks, which reportedly remain stable for several months. This behavior is unexpected, given the well-established role of ionomer in stabilizing catalyst dispersions, and therefore requires further explanation. Additionally, clarification is needed regarding whether conductive or stabilizing supports, such as ATO, were used in the ink formulations. These materials are commonly employed to enhance both dispersion stability and in-plane conductivity, especially at low Ir loadings (around 0.5 mg/cm^2 or lower). This point is also relevant because several of the observed effects such as the extent and mitigation of inactive regions, may differ substantially when using supported catalysts like Ir/ATO or similar. A discussion on whether the reported behavior persists in the presence of partially conductive supports would significantly strengthen the manuscript.

Response to (1a): stability of the ionomer-free IrO_2 ink.

The ionomer-free IrO_2 ink is dilute, with a solid-to-solution ratio of 0.38 wt%. At such a low particle concentration, the collision frequency between IrO_2 agglomerates is low, leading to slow agglomeration kinetics and no significant sedimentation over one month. Similar behavior has been reported for dilute catalyst dispersions (*e.g.*, ACS Appl. Mater. Interfaces 2019, 11, 45068). The measured zeta potential of the ionomer-free ink is –32 mV, which provides a moderate electrostatic repulsion that further suppresses compact aggregation. This level of ink stability is sufficient for the reproducible fabrication of uniform membrane electrode. We have added a concise discussion of this point in the revised manuscript (**Caption of Supplementary Fig. 17**).

Response to (1b): whether supports were used in the ink formulation?

No conductive or stabilizing supports were used in any of the catalyst layers studied in this work. This was an intentional choice to exclude the support effects and to focus on the intrinsic influence of the ionomer addition sequence. This point is stated in the revised manuscript (**Page 17**). For supported catalysts, the system is substantially more complex because the support's intrinsic conductivity, the catalyst-support interface, and the inter-particle contacts between supported catalysts all affect the in-plane electron transport. A systematic investigation of supported systems will be part of our future work.

(2) The study employs an *in situ* visualization technique to observe bubble evolution within the PTL and at the catalyst layer interface. While experimentally elegant and informative, the manuscript should more clearly define what phenomena are actually captured by this system. In some parts of the text, see for instance the caption to Figure 2 and lines 100-101 stating that "the electrochemical reaction in operating PEMWE was investigated using a self-built visualization system", the authors suggest that electrochemical reactions are directly visualized during operation; however, the images and videos provided appear to show only bubble formation and growth, not the reaction at catalytic active sites. It is well understood that most oxygen generated at catalytic sites initially dissolves into the electrolyte before forming bubbles, and thus these observations should not be interpreted as direct visualization of the reaction itself. Clarification is also needed regarding the significance of highlighted regions in the images (such as colored spots or contours) and how these relate to bubble dynamics.

Response 2: We thank the Reviewer for this careful and valuable suggestion. We agree that what is directly observed in our visualization system is the bubble evolution process, not the electrochemical reaction at the catalytic active sites. The original wording was imprecise and may have led to misinterpretation.

In our experiments, we use the spatial distribution of bubble nucleation sites as a proxy for the distribution of electrochemically active regions. This is because bubbles nucleate preferentially at locations where the dissolved gas concentration first exceeds the supersaturation threshold. That is, near the catalytically active sites where oxygen is generated. Therefore, while we do not directly visualize the reaction itself, the bubble distribution patterns provide indirect but meaningful information on the spatial distribution of catalytic activity within the PTL.

We have carefully revised the relevant text throughout the manuscript, including the caption of Figure 2 and the statements in the main text, to replace phrases such as "the electrochemical reaction … was investigated" with more precise descriptions, such as "the bubble nucleation sites … was monitored" or similar formulations. These revisions ensure that the scope and interpretation of the visualization data are accurately conveyed.

(3) The results presented here indicate that ionomer incorporation during deposition can lead to electronically insulated regions that reduce catalyst utilization. However, the

claim that the sequential method “activates previously inactive regions” is not yet fully supported by the presented evidence, since the catalytic activity (or lack of thereof) in any region has not been established for any system. The conductivity measurements indicate improvements, but more direct or quantitative evidence of increased catalyst utilization would be necessary, or alternatively the claim should be reconsidered accordingly.

Response 3: In our experiments, the spatial distribution of bubble nucleation sites was used as a proxy for the distribution of electrochemically active regions. For the one-step catalyst layer, oxygen bubbles were generated exclusively within a narrow zone adjacent to the PTL. In contrast, for the sequentially sprayed layer, bubble nucleation sites emerged at both the edges and the middle of the openings in the porous transport layer. These results directly confirm that the sequential fabrication method activates previously inactive regions.

We further evaluated iridium utilization by Cyclic voltammetry (CV) (**Figure R5 or Supplementary Fig. 22**). The sequentially sprayed anode layer exhibits a 36.7% larger CV area compared to the one-step sprayed anode layer. This result is consistent with the observed increase in activity, suggesting that the enhanced exposure of active sites due to the reactivation of electrochemically inactive zones is the primary factor for the improved activity.

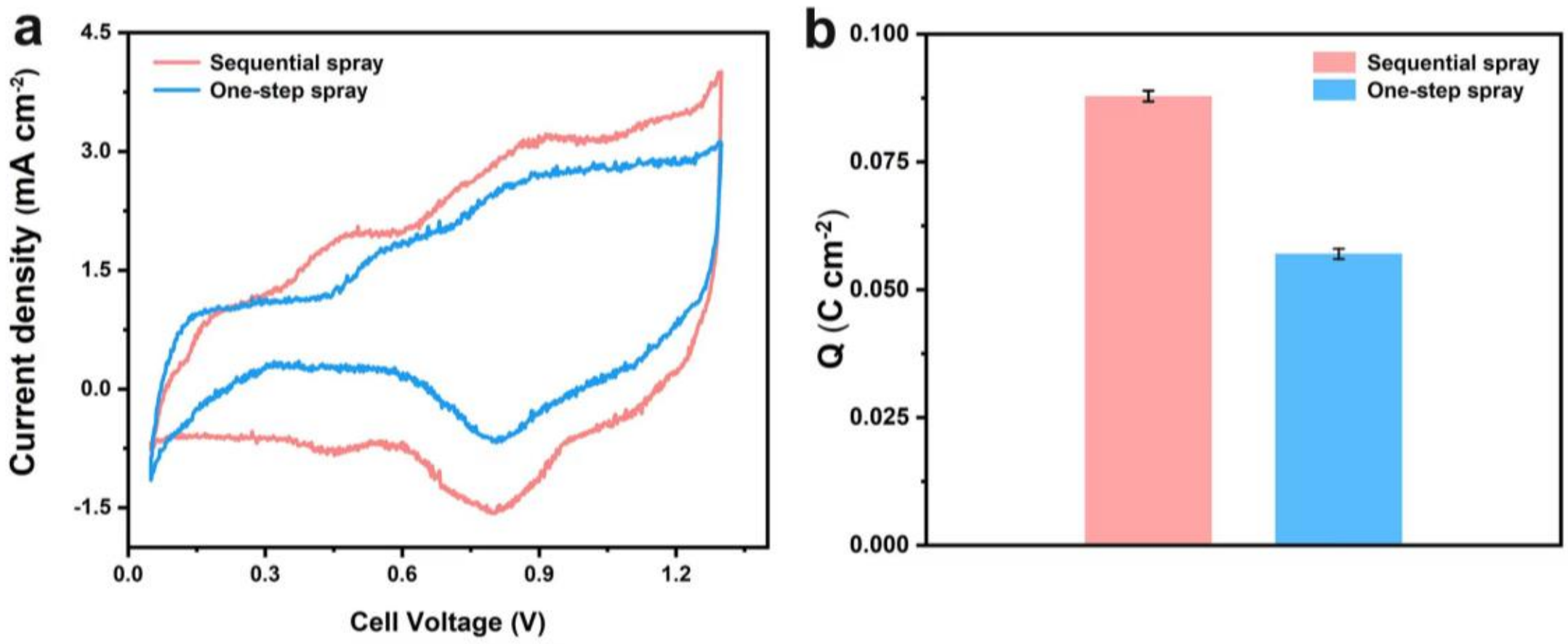

Figure R5 (a) Cyclic voltammetry (CV) curves for sequentially sprayed anode layer and one-step sprayed anode layer. (b) The pseudocapacitive charge (Q) values for sequentially sprayed anode layer and one-step sprayed anode layer calculated from CV curves.

(4) Also, it is important to consider the representativeness of the actual setup used for the visualization system. The highly macroporous PTLs used are ideal for enabling optical access, but both their morphology and porous structure differ substantially from the PTLs typically used in commercial and industrial PEMWE systems. The manuscript should therefore discuss the extent to which the visual observations can be extrapolated to conventional PTLs, and the limitations this may impose on the generality of the findings.

Response 4: We have tested the sequentially sprayed layer with both the STP-PTL (**Figure 4f**) and a conventional titanium fiber felt (**Figure 4a-c**) in cells. In both cases, the performance improvement over the one-step sprayed layer was comparable. This consistency confirms that the visual observations are relevant and that the strategy is scalable to practical PTL structures. This point is stated in the revised manuscript (**Page 14**).

Beyond this, we emphasize that the PTL structure does not affect our fundamental understanding of the origin of the "dead zones" or the validity of the proposed activation strategy. The underlying mechanism that ionomer disrupts the conductive network of IrO_2 nanocatalysts and creates in-plane dead zones is general, and the strategy of decoupling the ionomer from the catalyst layer to activate these zones remains broadly applicable. Only the specific numerical threshold required for in-plane activation to be re-evaluated when the PTL structure changes. This point is stated in the revised manuscript (**Page 8**).

(5) Several experimental aspects also require greater clarity. It is recommended that operating conditions (such as voltage) be stated explicitly in the relevant figures (*e.g.*, Figures 5a and 5c). The degradation behavior shown for the one-step sample in Figure 5d appears unusually rapid for IrO_x-based catalysts; clarification regarding the reproducibility of this result and whether it represents typical behavior would be valuable. Furthermore, the comparison of ionomer distribution between the one-step and sequential samples raises questions: the absence of a fluorine-rich overlayer in the sequential case is counterintuitive, and a more detailed explanation of ionomer penetration and distribution within the catalyst layer would improve the mechanistic narrative.

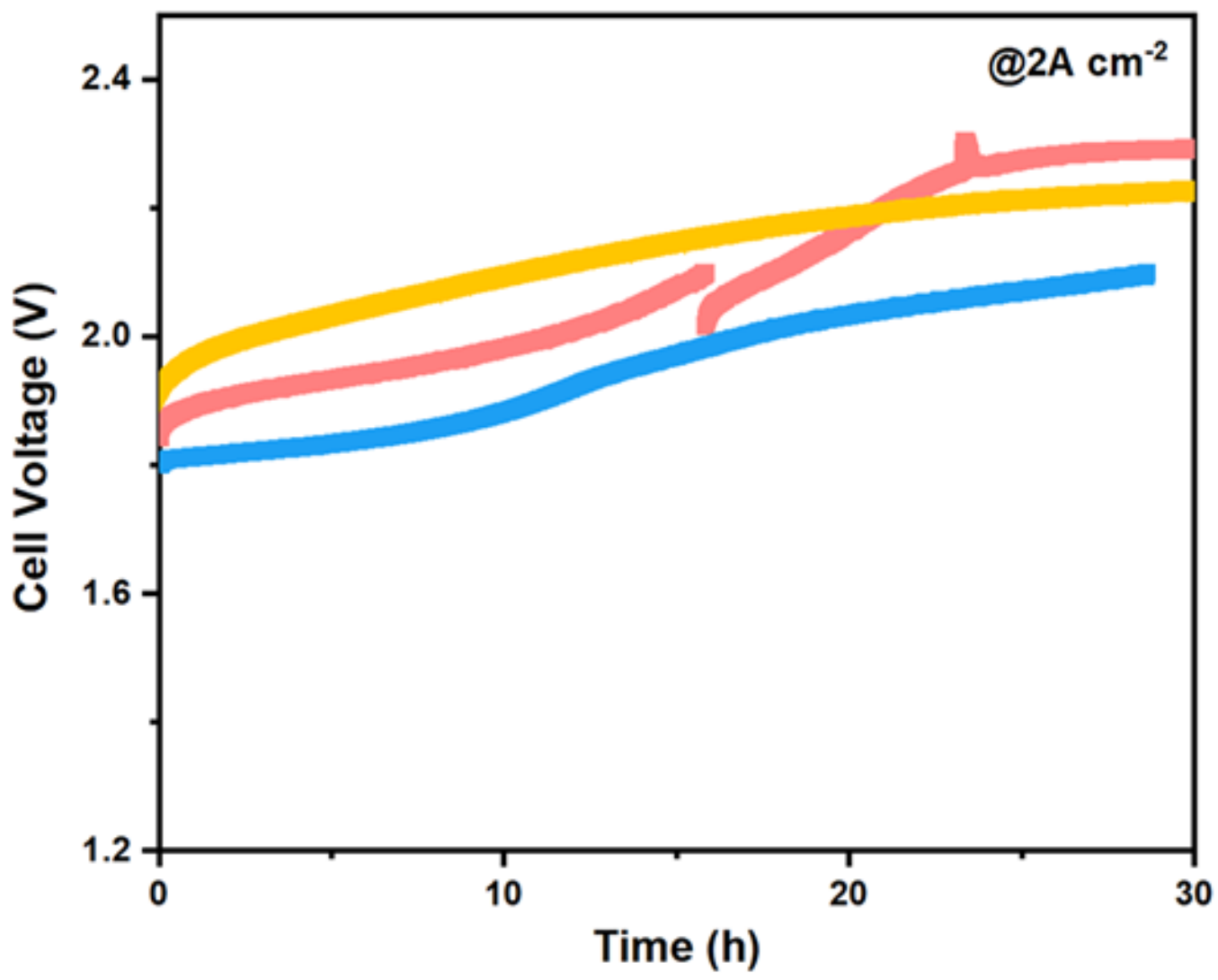

Figure R6 Chronopotentiometry curves of PEMWEs using one-step sprayed layer as anode under the Ir loading of 0.25 mg_{Ir} cm^{-2} at 1 A cm^{-2} current densities. Three independent replicate tests are shown, confirming the reproducibility of the rapid degradation behavior.

Response to (5a): operating condition and degradation reproducibility.

We thank the reviewer for these practical suggestions. We have now explicitly stated the operating conditions in Figures 5a and 5c (*i.e.*, 80 °C, Nafion 115, 2.0 V).

Regarding the rapid degradation observed for the one-step sample in Figure 5d, we confirm that this behavior is reproducible across multiple independent tests (3 replicates, **Figure R6 or Supplementary Fig. 25**). This rapid degradation is a typical observation for low-Ir-loading catalyst layers fabricated by the conventional one-step sprayed method (*e.g.*, Adv. Mater. 2025, 37, 2507560; Nat. Commun. 2023, 14, 5119). This degradation arises from the aggravated islanding effect of IrO_2 nanoparticles under PEMWE operating conditions, where agglomeration and maturation of IrO_2 nano-aggregates reduce active site accessibility, create larger electrical dead zones, and obstruct gas/liquid transport channels. In contrast, at a high Ir loading of 2 $mg_{Ir}\ cm^{-2}$, the one-step sprayed layer exhibits excellent stability (see Supplementary Fig. 24 in *Nat. Commun.* 2023, 14, 5119). We have added a discussion of this point and the reproducibility data in the revised manuscript.

Response to (5b): ionomer distribution in the sequential sample.

Our sequential method first deposits a porous IrO_2 skeleton, and then an ionomer solution is sprayed on top. Because the pre-formed IrO_2 skeleton is highly porous, the ionomer solution readily permeates through the entire thickness of the catalyst layer (**Figure 4b,c**) rather than accumulating at the surface. Upon drying, the ionomer forms a distributed, conformal coating on the IrO_2 particles throughout the layer.

To prepare a high-loading catalyst layer (>0.25 $mg_{Ir}\ cm^{-2}$), the strategy can be readily extended by repeating the "catalyst spray/ionomer spray" cycle two or more times. Specifically, thin IrO_2 sublayers are sequentially deposited and infiltrated with ionomer in a stepwise manner to gradually build up the total catalyst loading. Because each individual sublayer remains thin, uniform ionomer penetration can still be maintained, ensuring effective ionomer distribution throughout the catalyst layer and efficient contact with the membrane interface. We have added a discussion of this point in the revised manuscript (**Page 17**).

(6) Finally, since the sequential deposition strategy is presented as potentially scalable, it would be beneficial for the manuscript to discuss its compatibility with industrially relevant coating processes such as doctor blading or roll-to-roll manufacturing in which ink viscosity, wettability, and uniformity are tightly coupled to ionomer content. Despite these points requiring clarification, the manuscript offers a meaningful conceptual advance and a promising experimental approach, and addressing the issues outlined above would significantly strengthen its impact.

Response 6: We agree that for a strategy proposed as scalable, discussing its compatibility with industrial coating processes is essential. We would like to clarify that the essential novelty of the sequential method lies not in the specific ink concentrations or spraying parameters used in our lab-scale demonstration, but in its fundamental design principle. It decouples the deposition of the pristine conductive IrO_2 skeleton from ionomer infiltration, thereby preserving continuous electron transport pathways

that are otherwise disrupted by ionomer in the one-step sprayed layer. This decoupling concept is generic and not inherently tied to ultrasonic spraying.

For industrially relevant processes such as doctor blading or roll-to-roll manufacturing, the same principle can be translated into a two-step coating sequence. For instance, a catalyst layer can be blade-coated from an ionomer-free ink optimized solely for film uniformity, porosity, and adhesion, while the ionomer can be introduced in a subsequent impregnation using a low-viscosity solution. This separation offers great flexibility for large-scale production. Thus, rather than being limited by the spraying method, the decoupling strategy provides a conceptual framework to guide the development of industrially viable processes. We have added a discussion of this point in the revised manuscript (**Page 16**).

Reviewer #3 (Remarks to the Author):

I co-reviewed this manuscript with one of the reviewers who provided the listed reports. This is part of the Nature Communications initiative to facilitate training in peer review and to provide appropriate recognition for Early Career Researchers who co-review manuscripts.

Response: We appreciate the time and effort you have devoted to evaluating our manuscript, and we are grateful for the constructive feedback provided jointly with the other reviewer. Your input has been carefully considered in our revision, and we hope the improved manuscript meets your expectations.
