# Supplementary material for: Activating in-plane dead zones of anode catalyst layers in proton exchange membrane water electrolyzers
Source: Nat Commun. 2026 Jul 1;17:8211. doi: 10.1038/s41467-026-75223-1 (PMC13462438; doi:10.1038/s41467-026-75223-1)
Supplement: Supplementary file 2 [file 41467_2026_75223_MOESM1_ESM.pdf]

Supplementary Information (SI) for

Activating In-Plane Dead Zones of Anode Catalyst Layers in Proton Exchange Membrane Water Electrolyzers

Zicheng Zhao,[†,1] Xiao Liang,[†,1] Nan Lin,[1] Zhenye Kang,[2] Yuchang Hou,[1] Yaoxin Wang,[1] Hui Chen,[*,1] Xiaoxin Zou[*,1,3,4]

[1] State Key Laboratory of Inorganic Synthesis and Preparative Chemistry, College of Chemistry, Jilin University, Changchun 130012, China

[2] Key Laboratory of Agro-Forestry Environmental Processes and Ecological Regulation of Hainan Province, State Key Laboratory of Tropic Ocean Engineering Materials and Materials Evaluation, Hainan Provincial Key Lab of Fine Chem, School of Environmental Science and Engineering, Hainan University, 58 Renmin Road, Haikou 570228, China

[3] Changbaishan Laboratory, Changchun 130012, China

[4] National Demonstration Center for Experimental Chemistry Education, Jilin University, Changchun 130012, China

* E-mail: chenhui@jlu.edu.cn; xxzou@jlu.edu.cn

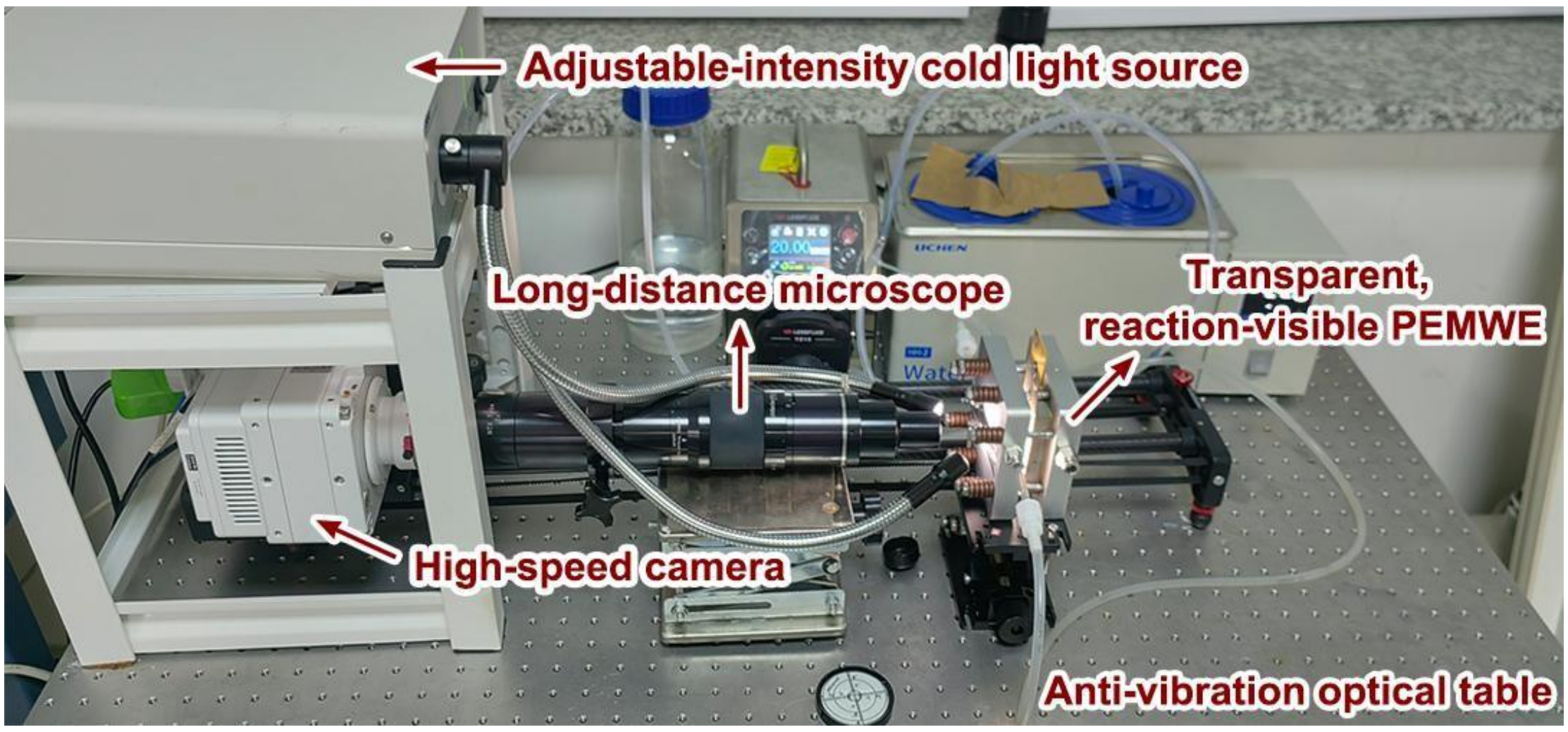

Supplementary Fig. 1 Digital photograph of a transparent and reaction-visible PEMWE equipped with a high-speed and microscale visualization system.

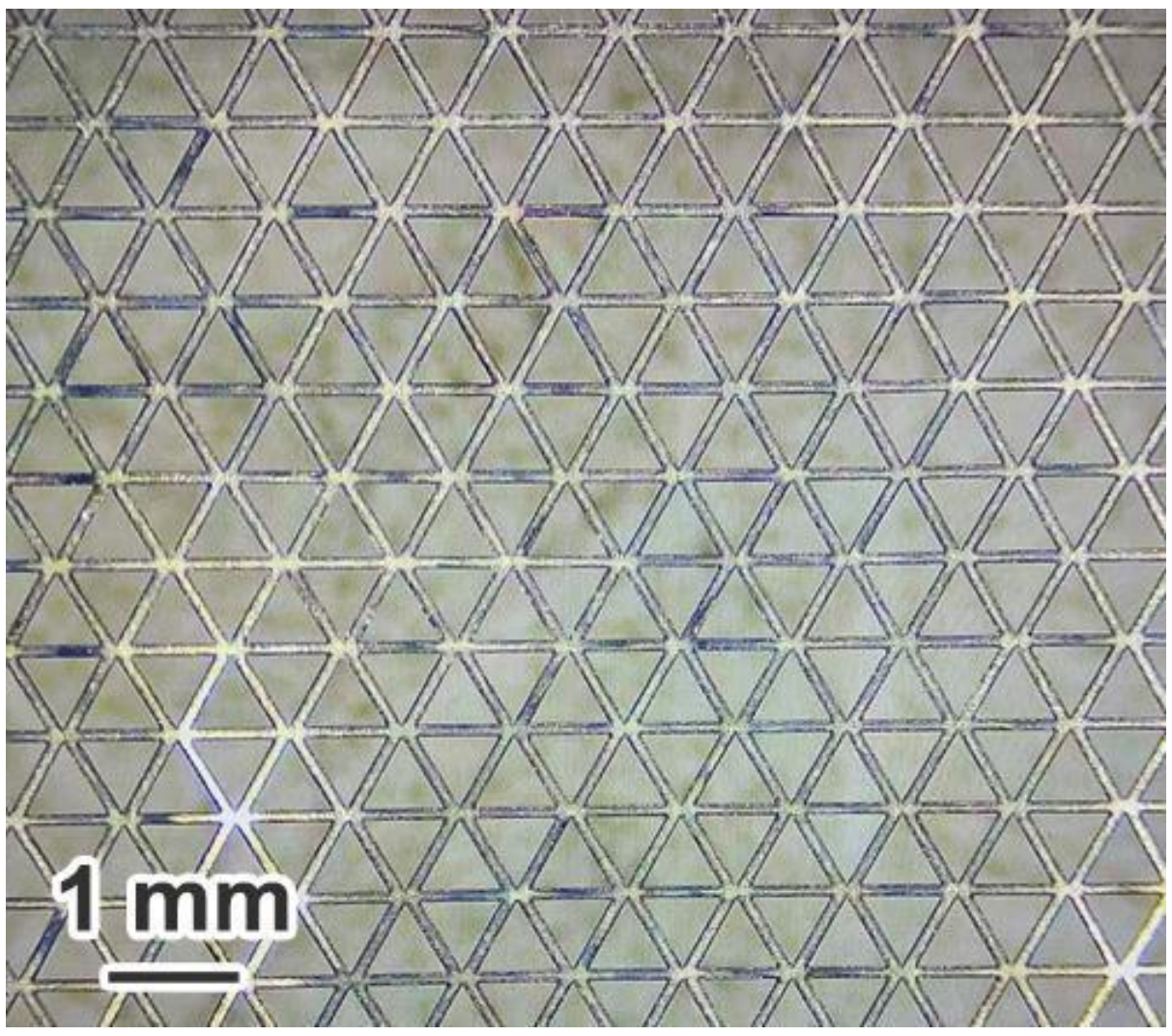

Supplementary Fig. 2 Digital image of the thin Ti-based porous transport layer with straight-through pores (STP-PTL).

The STP-PTL incorporates a uniform array of triangular apertures. This configuration provides an opening height of approximately 600 μm and an opening wall width of about 150 μm. The STP-PTL allows for direct optical access to the anode catalyst layer (ACL), enabling observation of the electrochemical reactions occurring within the microchannel.

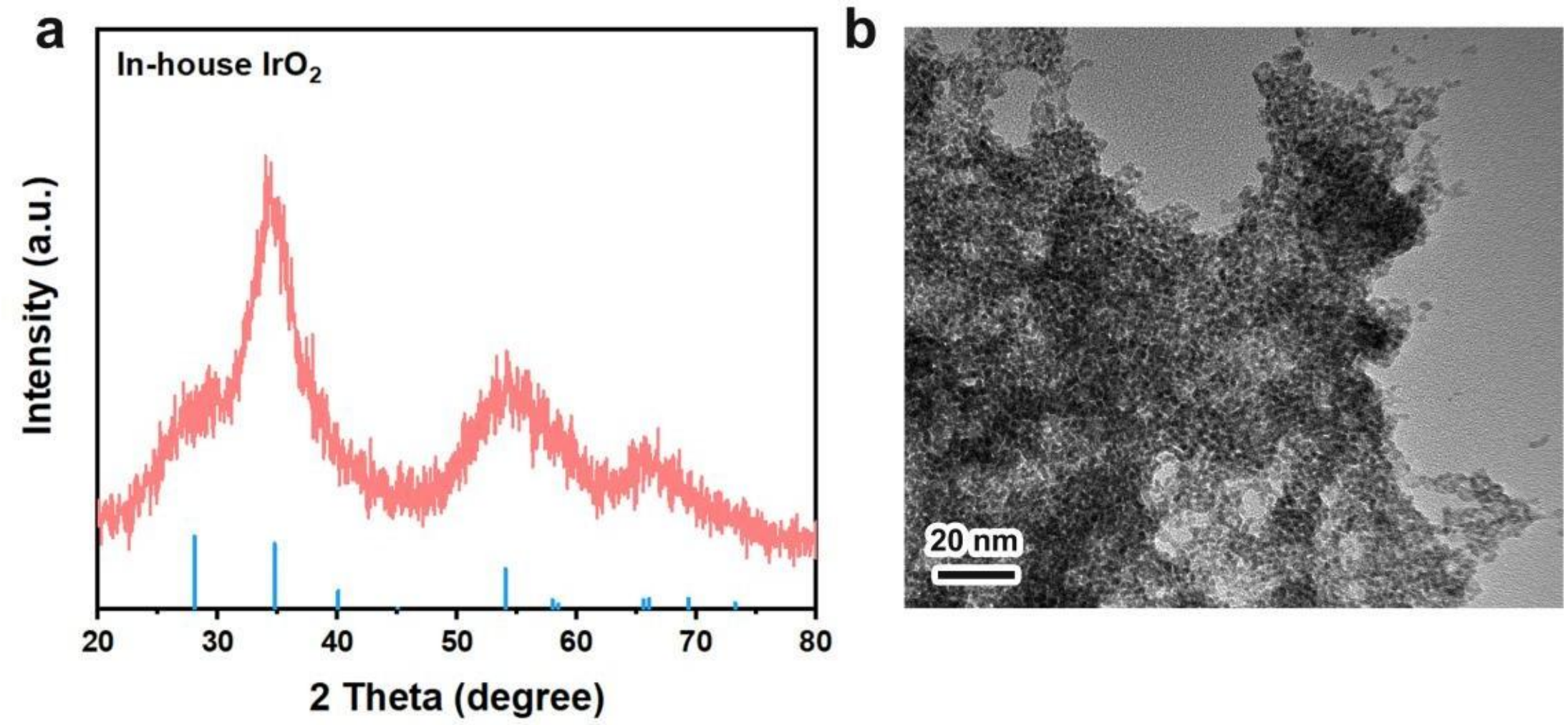

Supplementary Fig. 3 (a) XRD pattern and (b) TEM image of in-house synthesized IrO_2. The in-house synthesized IrO_2 comprises aggregates of circa 1.6 nm nanoparticles.

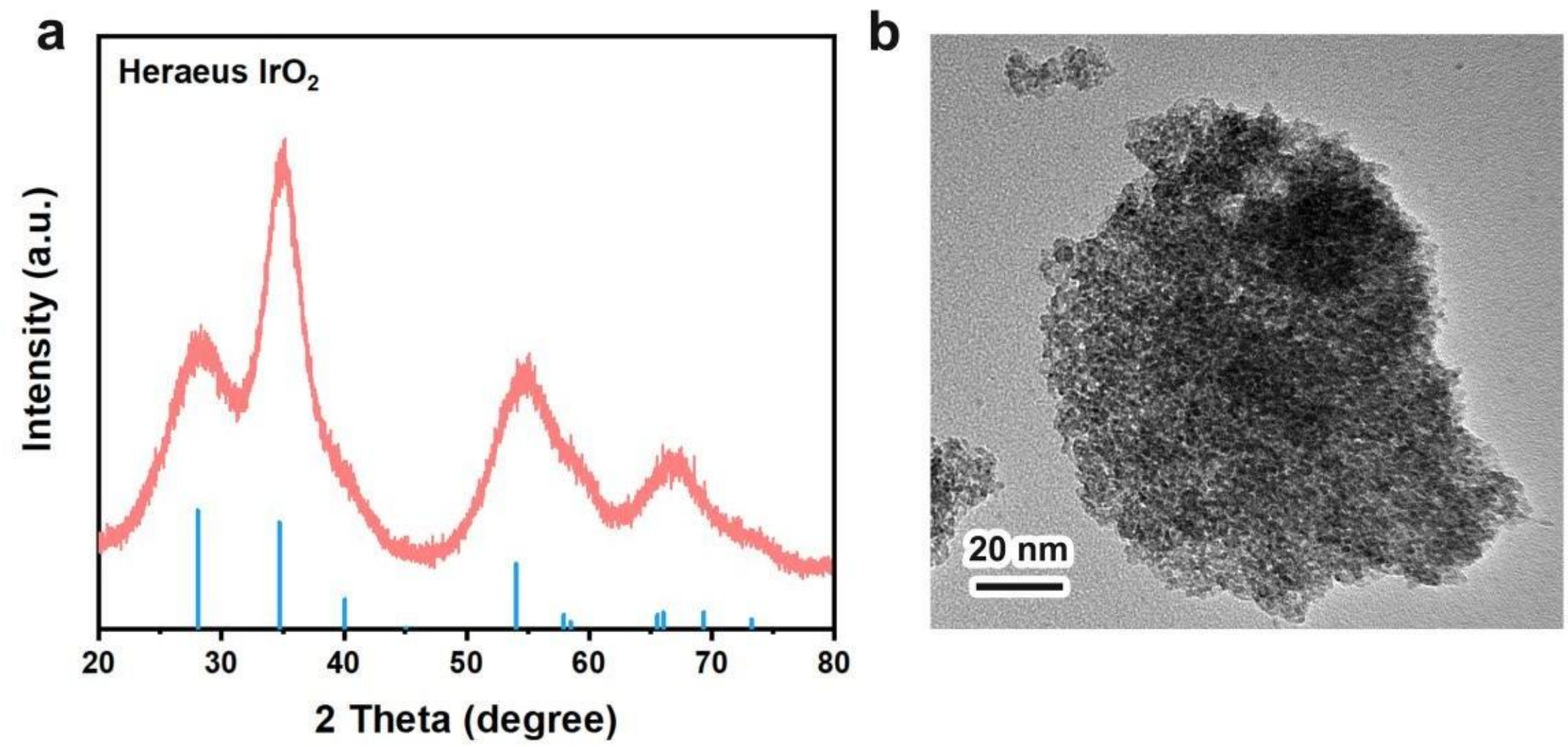

Supplementary Fig. 4 (a) XRD pattern and (b) TEM image of commercial IrO_2 from Heraeus (Germany). The Heraeus IrO_2 comprises aggregates of circa 2.3 nm nanoparticles.

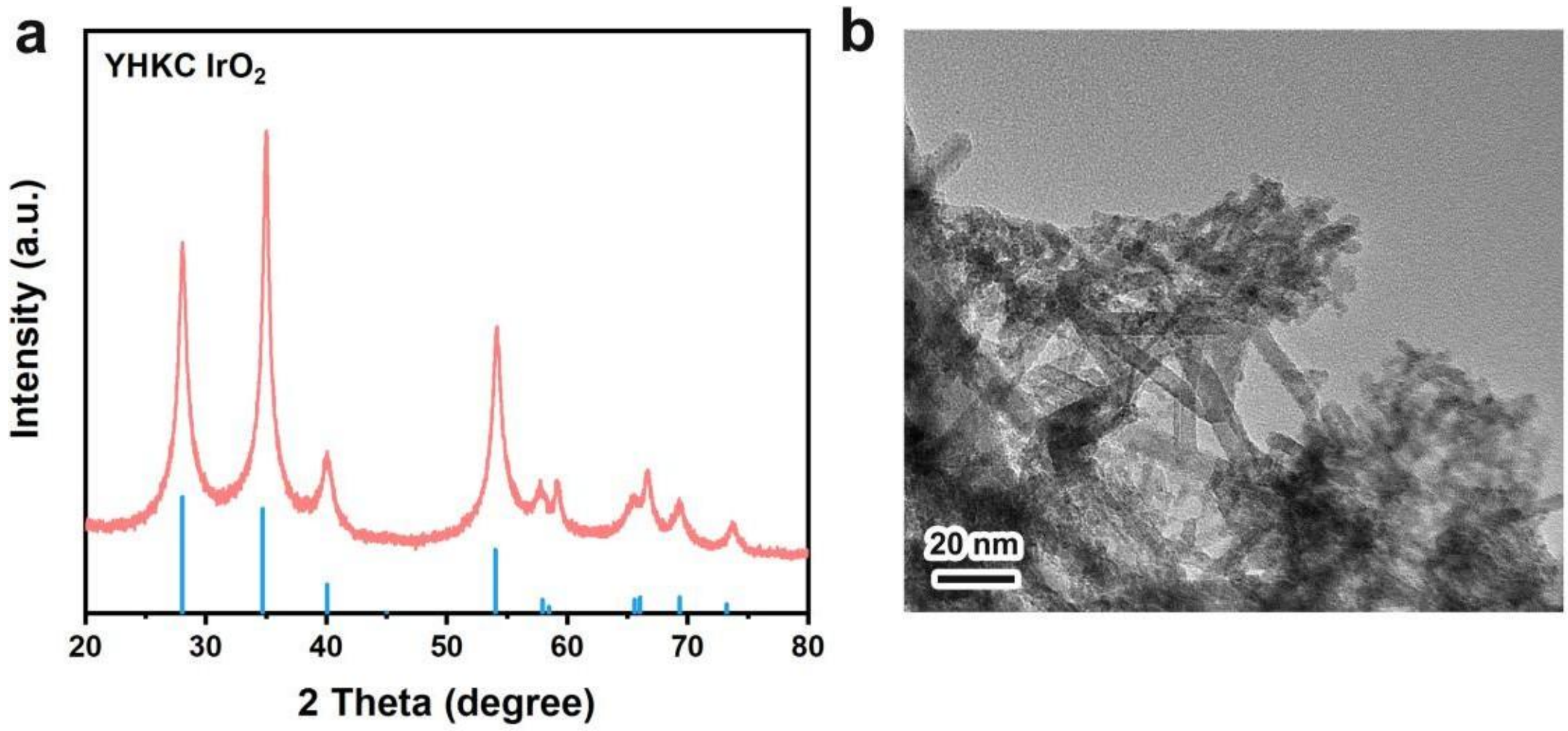

Supplementary Fig. 5 (a) XRD pattern and (b) TEM image of commercial IrO_2 from YHKC (China). The YHKC IrO_2 consists of nanorods with a circa width of 4.8 nm and a length on the order of tens of nanometers.

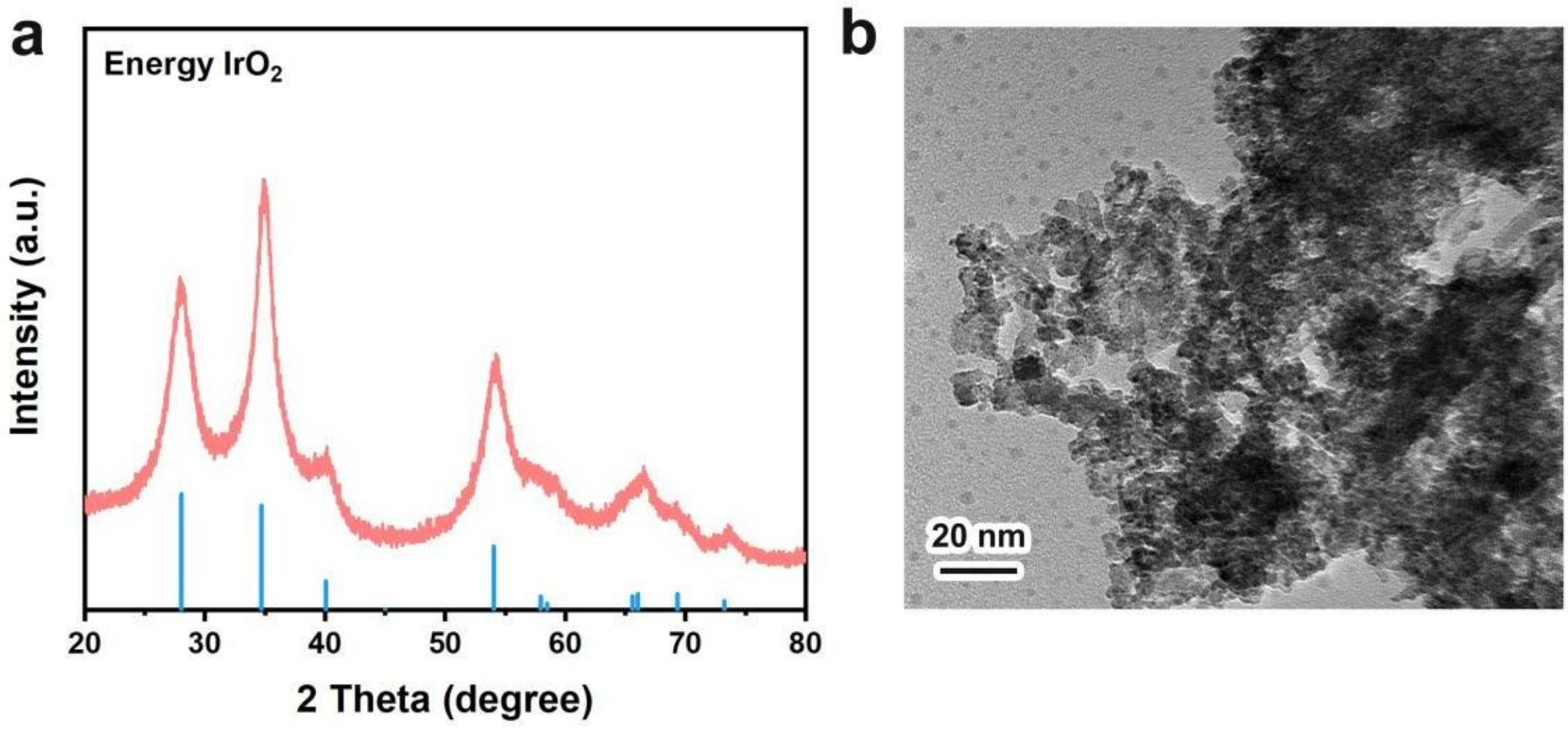

Supplementary Fig. 6 (a) XRD pattern and (b) TEM image of commercial IrO_2 from Energy (China). The Energy IrO_2 comprises aggregates of circa 3.7 nm nanoparticles.

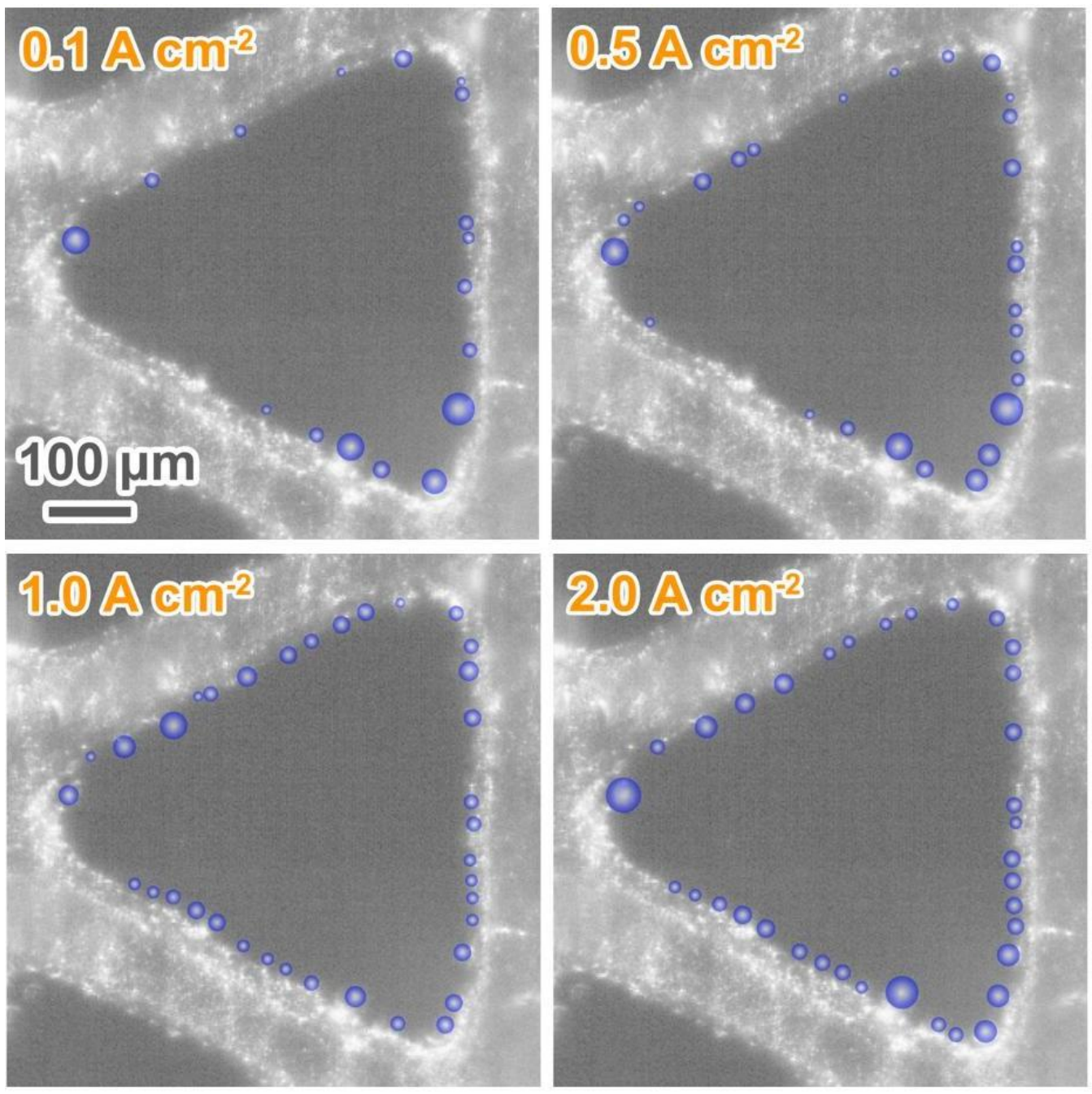

Supplementary Fig. 7 Front-view visualization of electrochemical reactions occurring within the microchannel of PEMWEs operating at current densities ranging from 0.1 to 2.0 A cm^{-2}.

We tested one-step sprayed anode layer across current densities from 0.1 to 2 A cm^{-2} by the self-built visualization system. The spatial distribution of electrochemical activity was found to be strictly confined to the edge of porous transport layer, regardless of the current density.

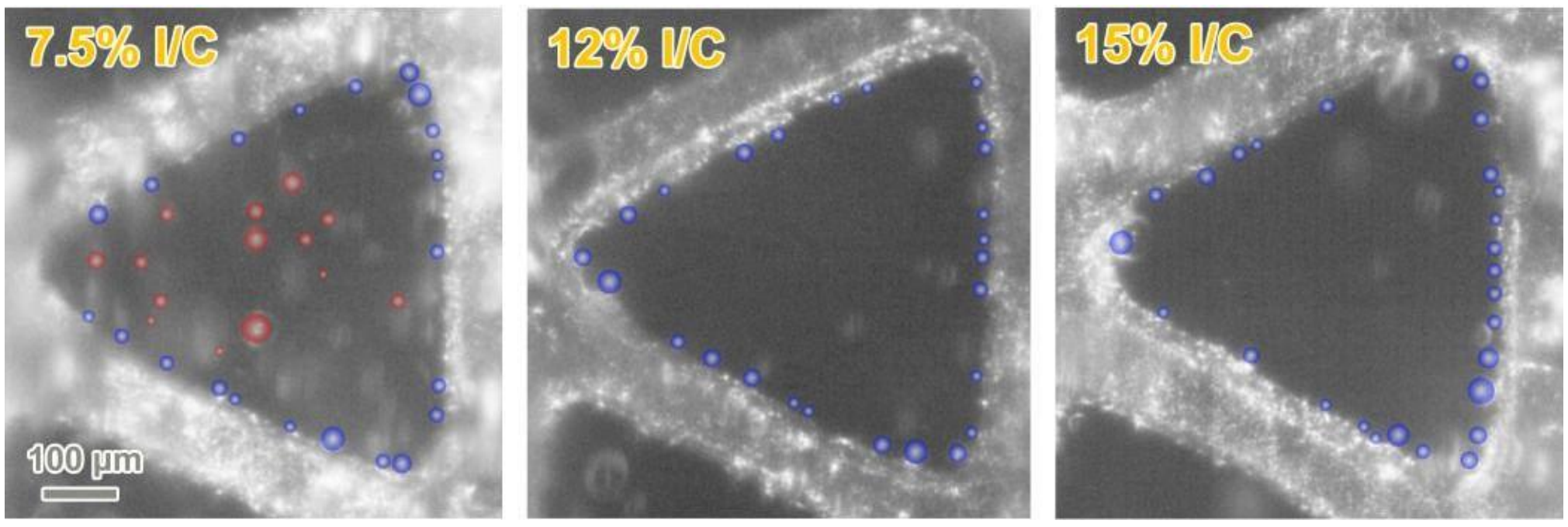

Supplementary Fig. 8 Front-view visualization of electrochemical reactions occurring within the microchannel of PEMWEs employing different ionomer/catalyst ratio (7.5%,12%, and 15%).

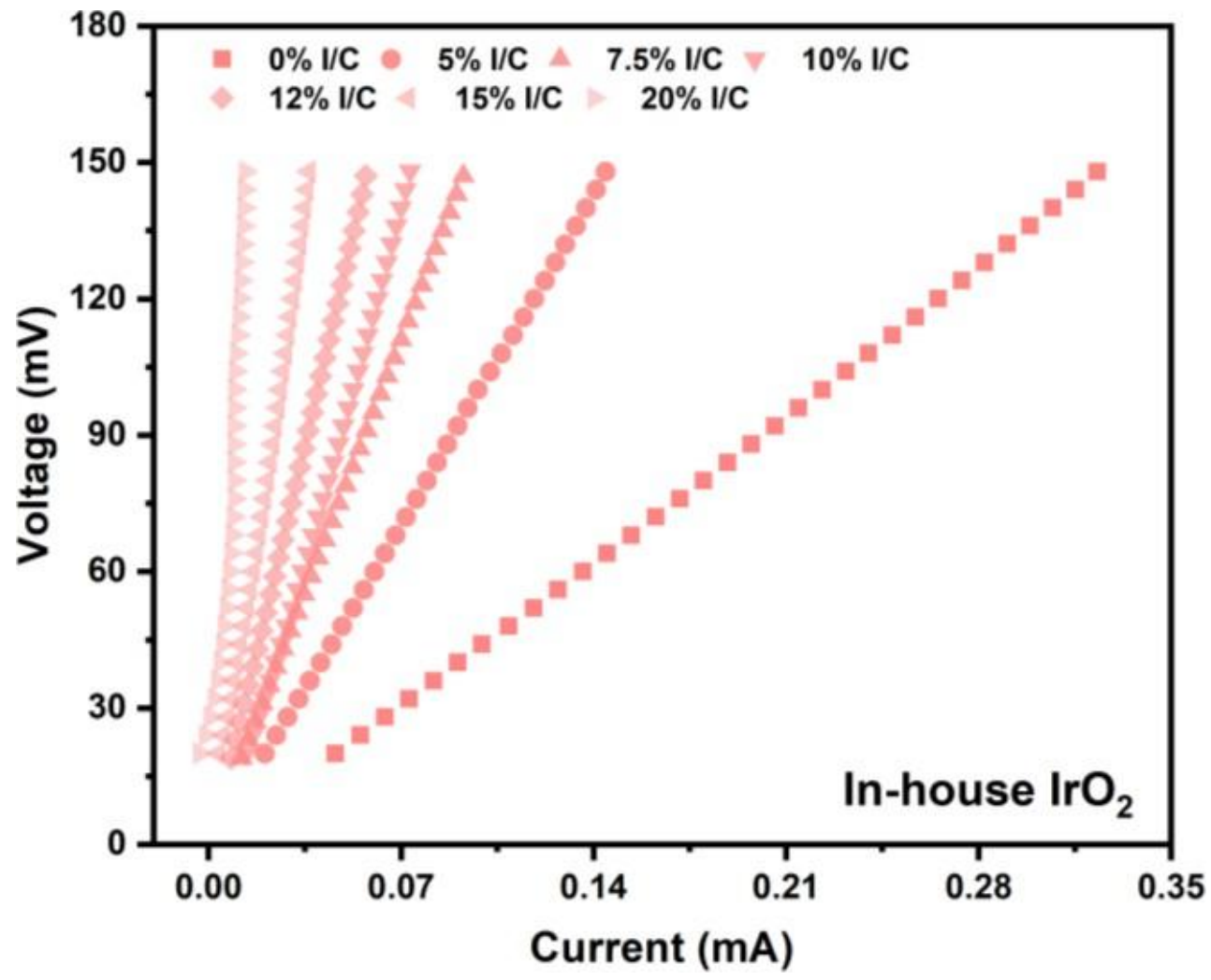

Supplementary Fig. 9 The in-plane electronic conductivity of catalyst layers with the ionomer/catalyst ratio from 0 to 20 wt% measured by the four-point probe method. These catalyst layers are fabricated with in-house synthesized IrO_2 at a constant iridium loading of 0.25 mg_{Ir} cm^{-2}.

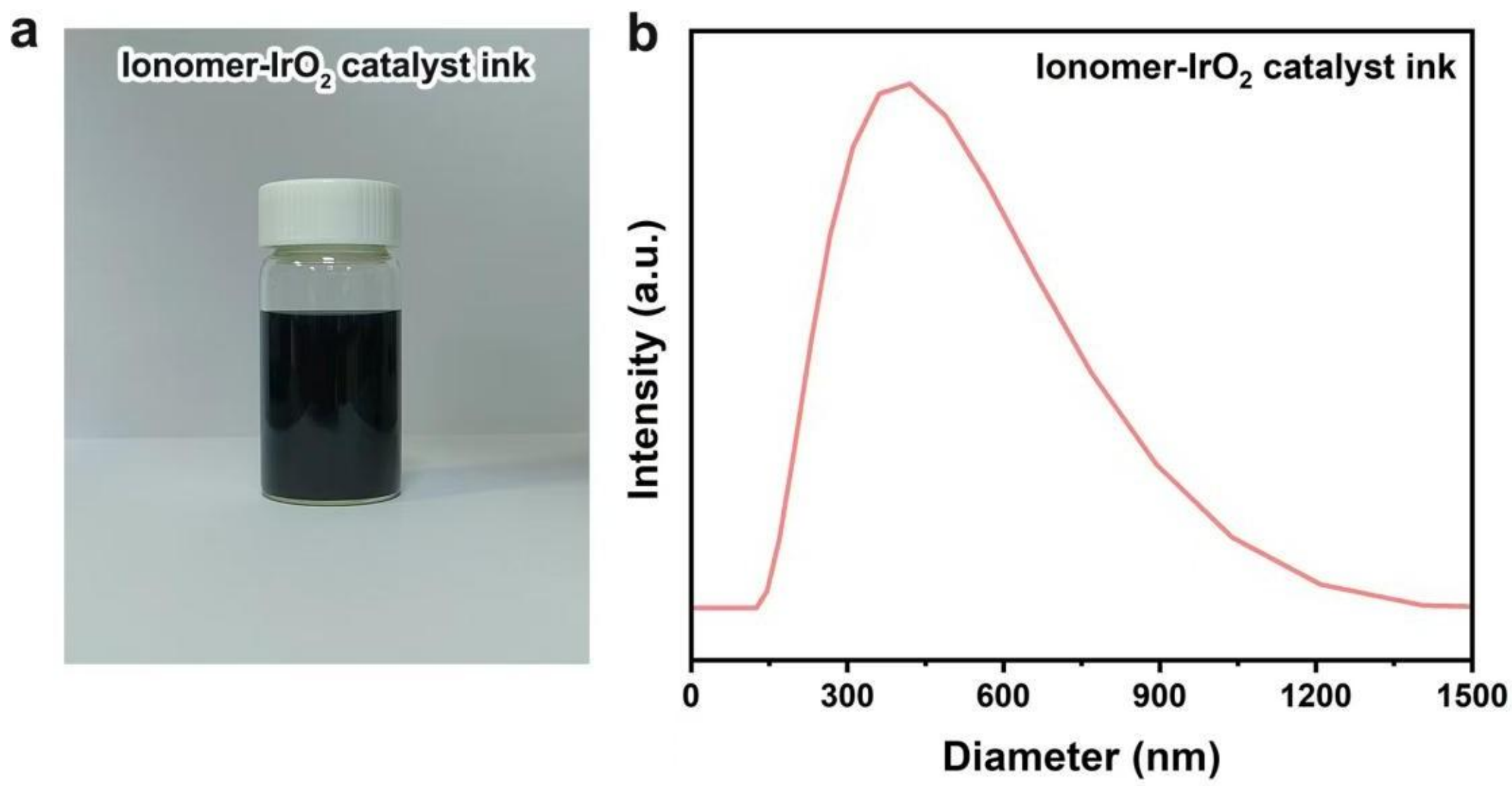

Supplementary Fig. 10 (a) Digital image and (b) dynamic light scattering (DLS) characterization of the ionomer-IrO_2 catalyst ink. The catalyst ink is fabricated with in-house synthesized IrO_2 with an ionomer/catalyst ratio of 15 wt%.

Zeta potential measurements of the catalyst ink yielded a strongly negative value of –36 mV. High dispersion stability of the catalyst ink can be attributed to significant electrostatic repulsion among the IrO_2-ionomer agglomerates.

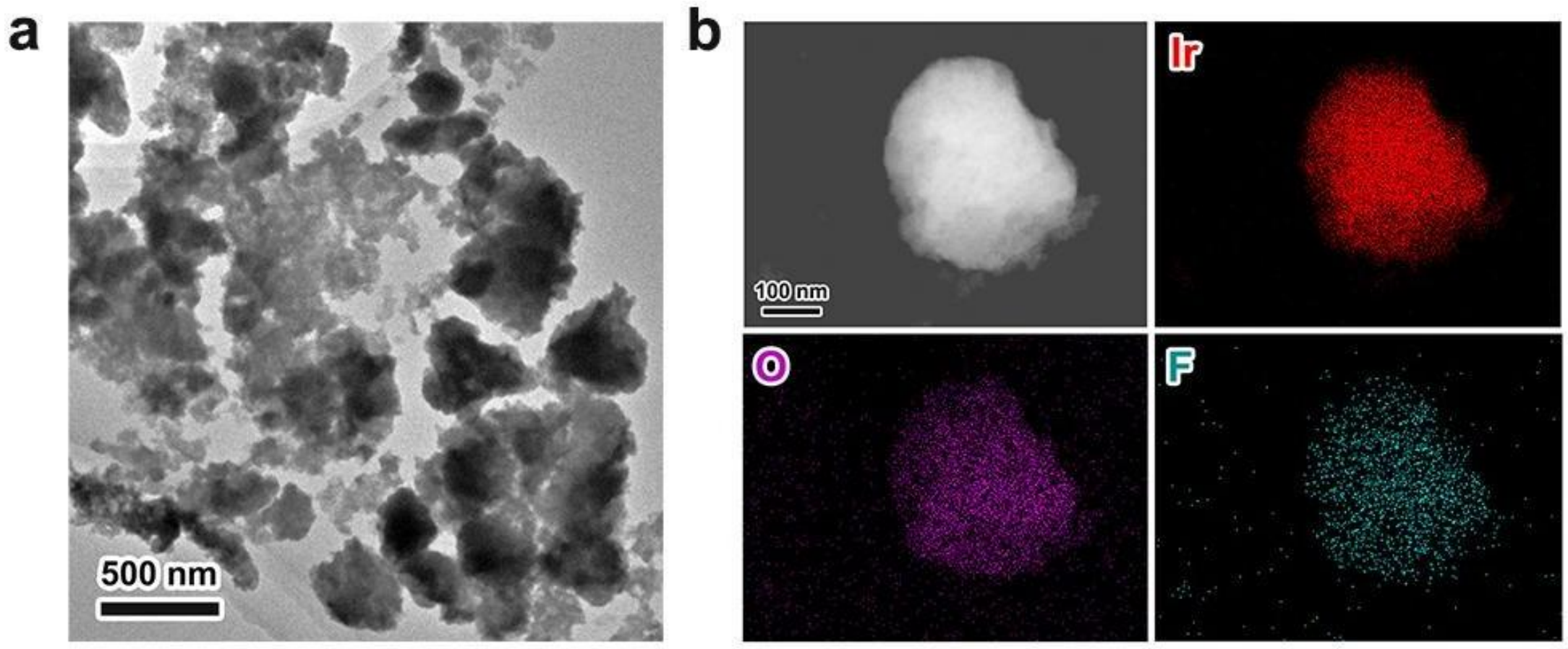

Supplementary Fig. 11 (a) TEM image and (b) corresponding elemental mapping images of the dried ionomer-IrO_2 catalyst ink. The catalyst ink is fabricated with in-house synthesized IrO_2 with an ionomer/catalyst ratio of 15 wt%.

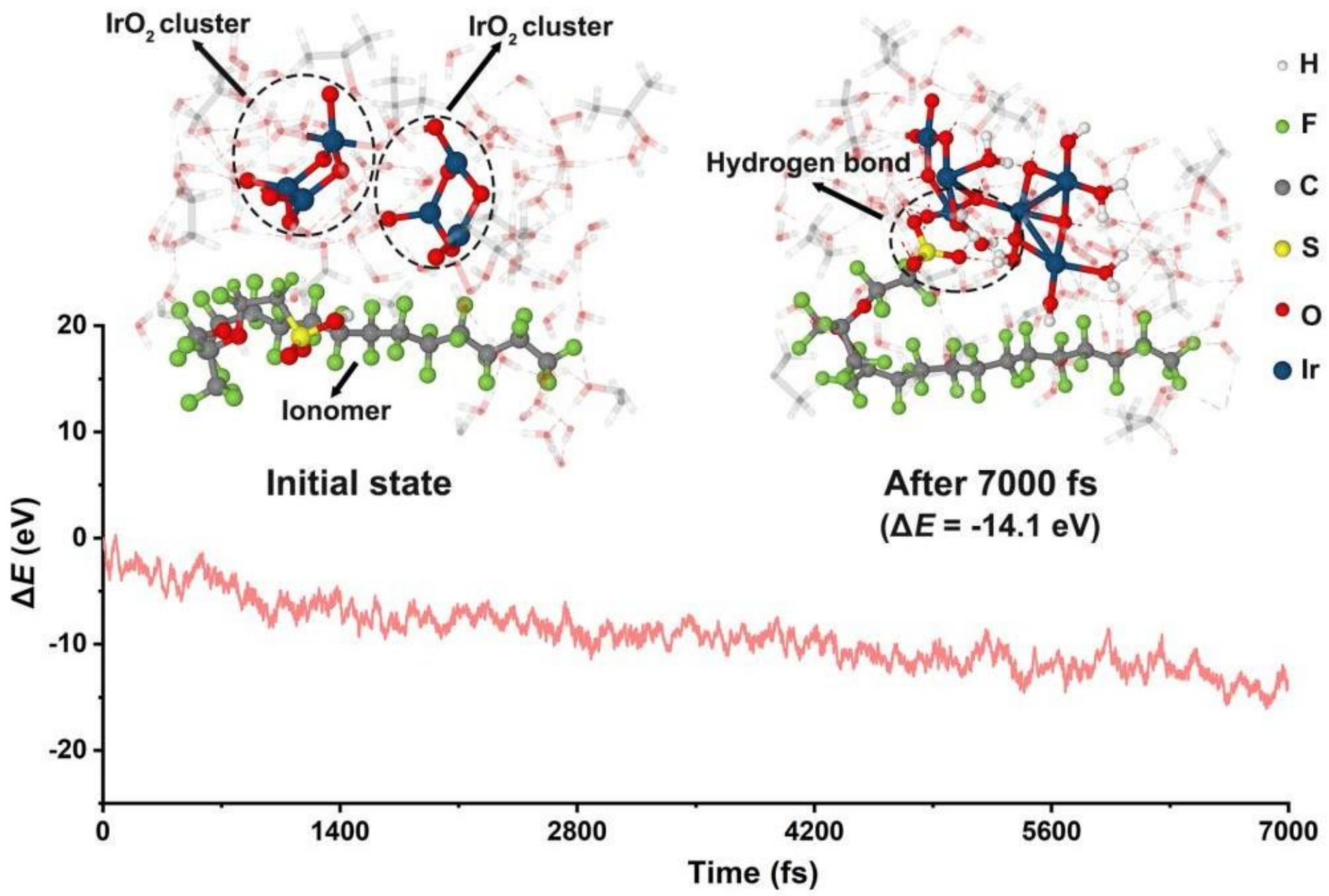

Supplementary Fig. 12 Molecular dynamics (MD) simulations of Nafion monomers and IrO_2 clusters dispersed in a water-isopropanol mixed solvent.

To gain molecular-level insight into catalyst particles-Nafion interactions, we performed molecular dynamics (MD) simulations of Nafion monomers and IrO_2 clusters dispersed in water: isopropanol = 8:1 mixture solvent. The structure at 7000 fs reveals the formation of hydrogen bonds between the IrO_2 clusters and the sulfonate groups of the Nafion monomers.

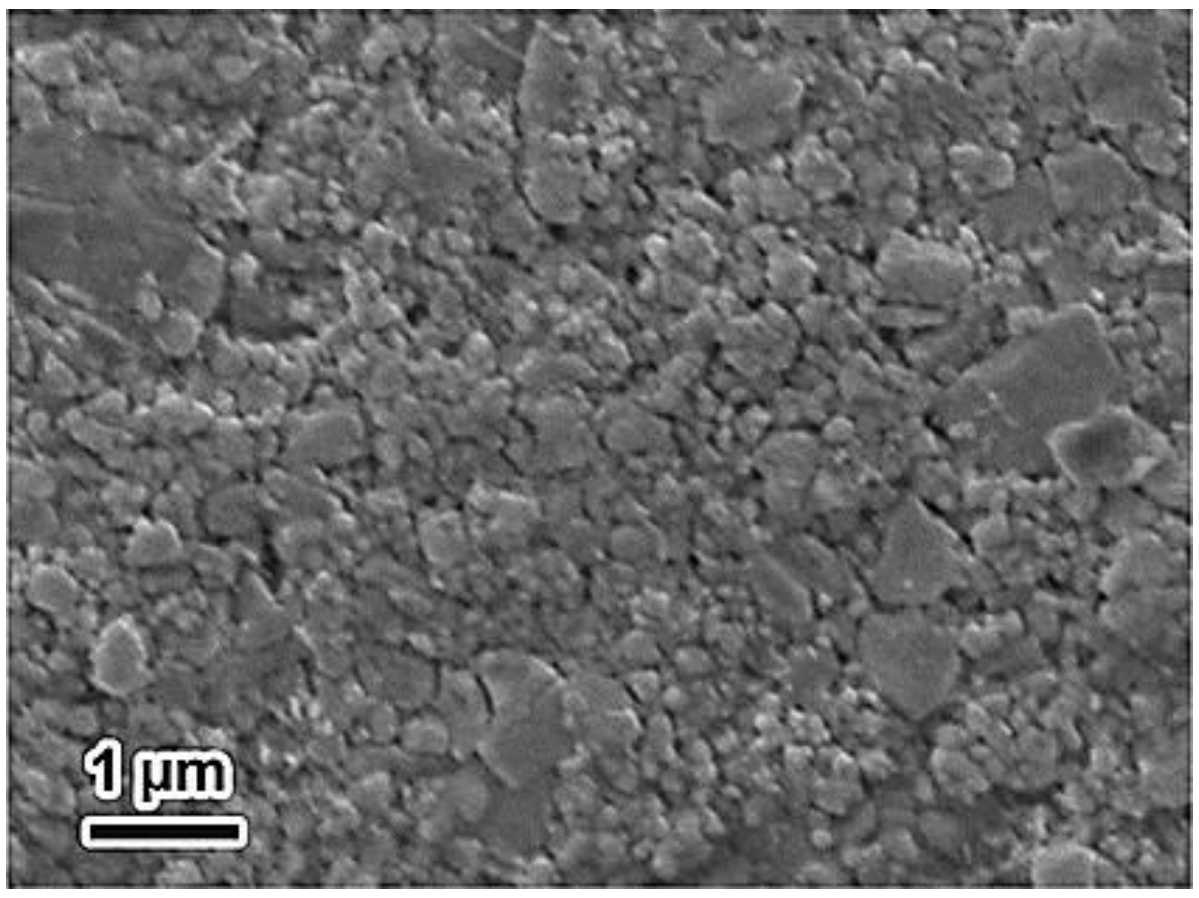

Supplementary Fig. 13 SEM image of one-step sprayed anode layer. The catalyst layer is fabricated with in-house synthesized IrO_2 at an iridium loading of 1 mg_{Ir} cm^{-2}.

The one-step sprayed anode layer is composed of IrO_2-ionomer agglomerates on the scale of hundreds of nanometers, in good agreement with the TEM results of the catalyst ink in **Supplementary Fig. 11**.

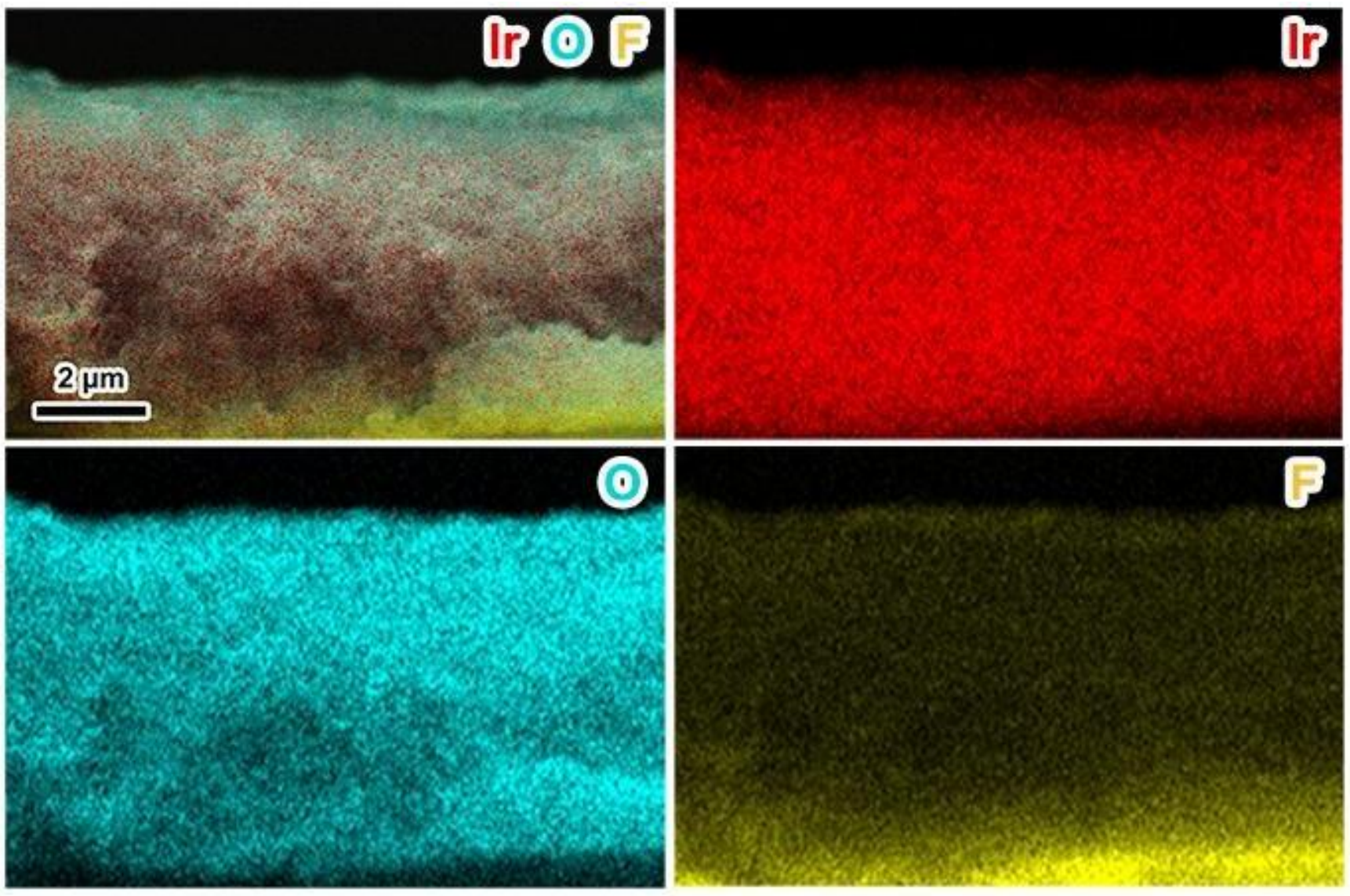

Supplementary Fig. 14 Elemental mapping images of Ir, O, and F in the one-step sprayed anode layer. The catalyst layer is fabricated with in-house synthesized IrO_2 at an iridium loading of 1 mg_{Ir} cm^{-2}. The F element (a signature element of Nafion) is uniformly distributed over the entire anode layer.

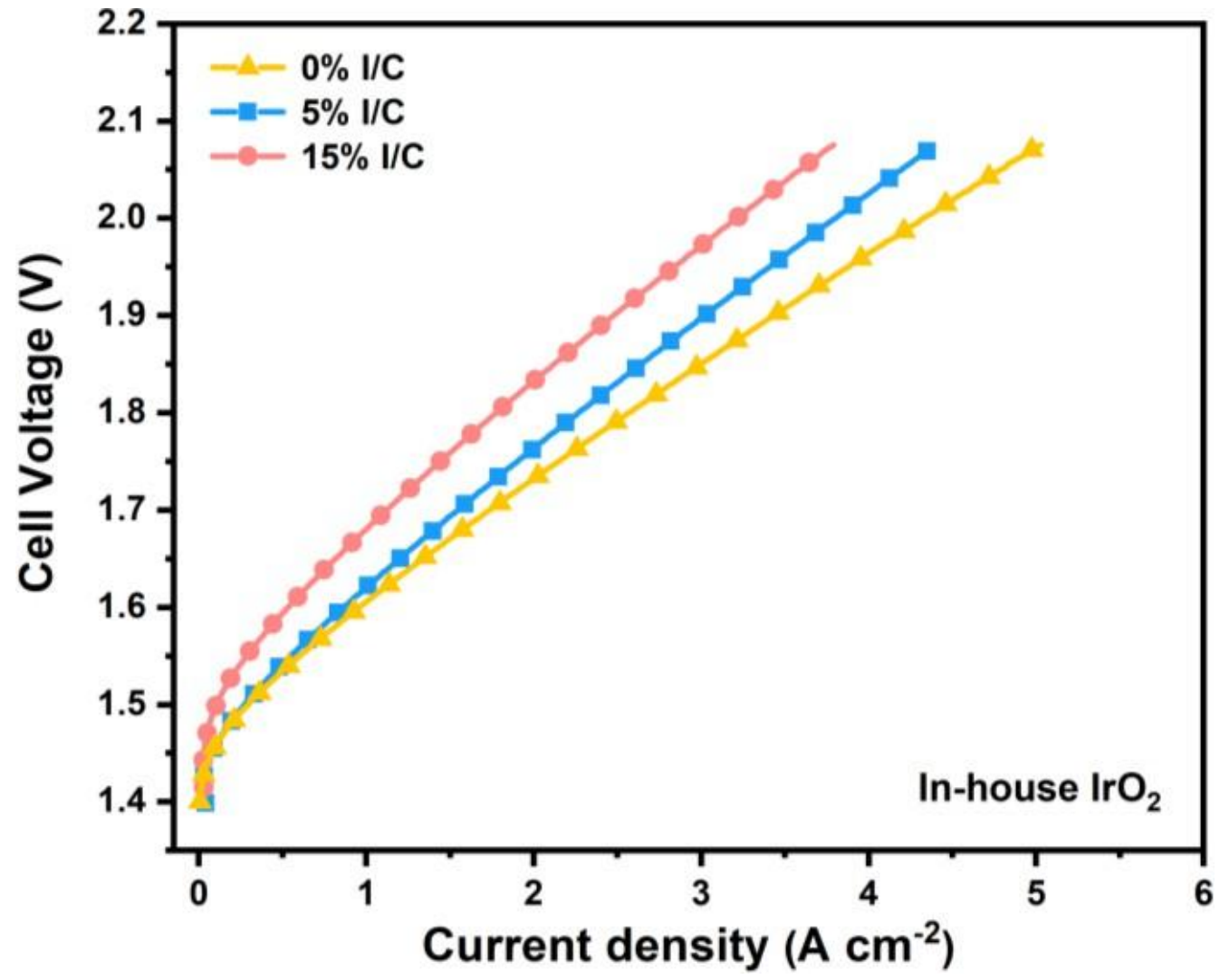

Supplementary Fig. 15 Steady-state polarization curves of PEMWE using one-step sprayed layers as anodes with 0 wt%, 5 wt% and 15 wt% ionomer/catalyst (I/C) ratios (80 °C, Nafion 115). These catalyst layers are fabricated with in-house synthesized IrO_2 at a constant iridium loading of 0.25 mg_{Ir} cm^{-2}.

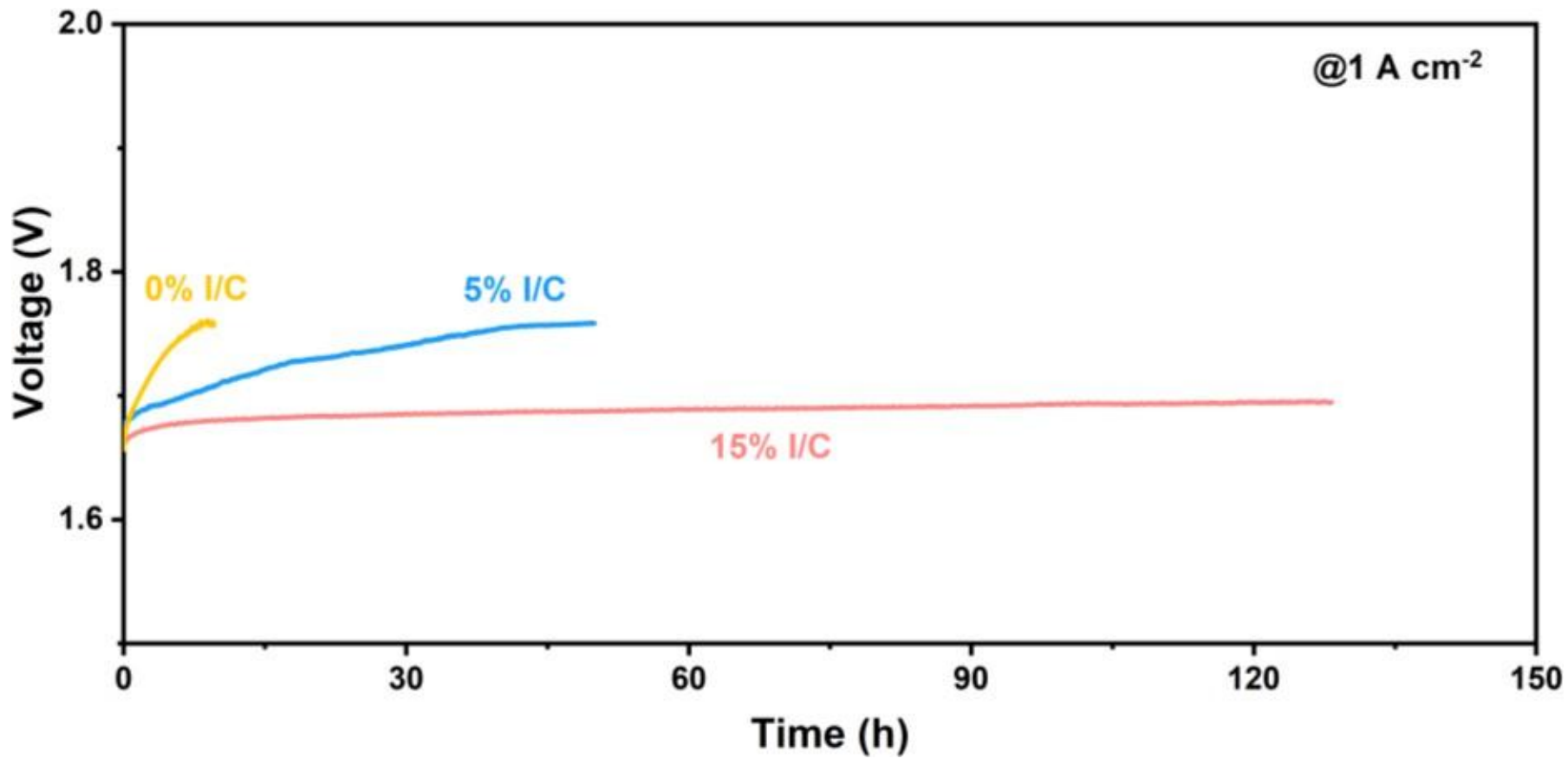

Supplementary Fig. 16 Chronopotentiometry curves of PEMWEs using one-step sprayed layers as anodes with 0 wt%, 5 wt% and 15 wt% ionomer/catalyst ratios.

While reducing ionomer content in one-step sprayed layer improves activity (**Supplementary Fig. 15**) by enhancing the in-plane electronic conductivity, it inevitably sacrifices stability (**Supplementary Fig. 16**). This degradation originates from increased IrO_x detachment and disrupted proton-conducting pathways caused by reduced ionomer content. This inherent trade-off makes it challenging to simultaneous optimization of activity and durability for conventional one-step sprayed layer.

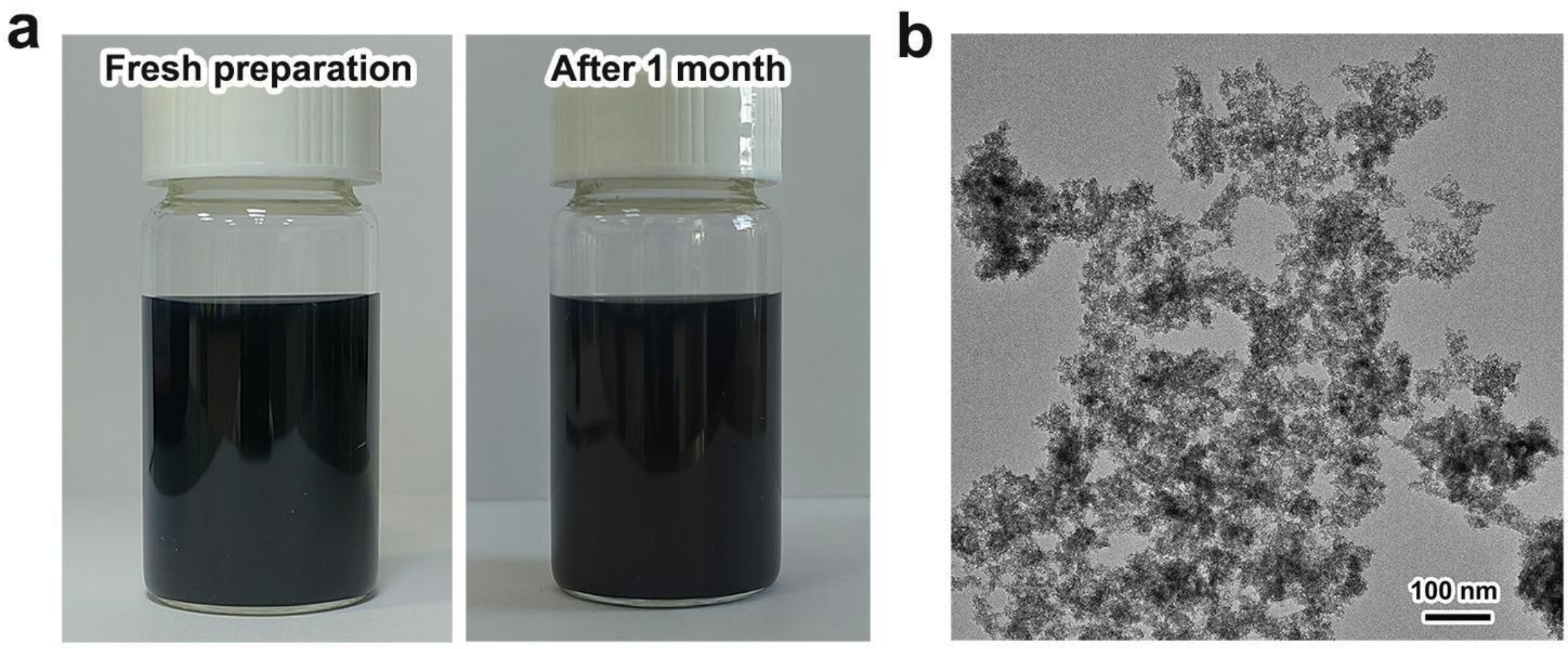

Supplementary Fig. 17 (a) Optical photographs of the dried, ionomer-free IrO_2 catalyst ink in the freshly prepared state and after one month of storage. (b) TEM image of the dried, ionomer-free IrO_2 catalyst ink.

The ionomer-free IrO_2 ink exhibits excellent dispersion stability, with no observable sedimentation after one month of storage (**Supplementary Fig. 17a**). TEM characterization of the dried ink (**Supplementary Fig. 17b**) reveals a distinct morphological characteristic: the IrO_2 nanoparticles self-assemble into a loose nanonetwork, in contrast to the dense IrO_2-ionomer agglomerates typically formed in the presence of ionomer. This observation can be attributed to two key factors. First, the absence of ionomer removes the strong ionomer-IrO_2 interactions that that would otherwise promote compact aggregation. Second, the ionomer free IrO_2 ink is dilute, with a solid-to-solution ratio of 0.38 wt%. At such a low particle concentration, the collision frequency between IrO_2 agglomerates is low, leading to slow agglomeration kinetics. The measured zeta potential of the ionomer-free ink is -32 mV, which provides a moderate electrostatic repulsion that further suppresses compact aggregation. This level of ink stability is sufficient for the reproducible fabrication of uniform membrane electrode.

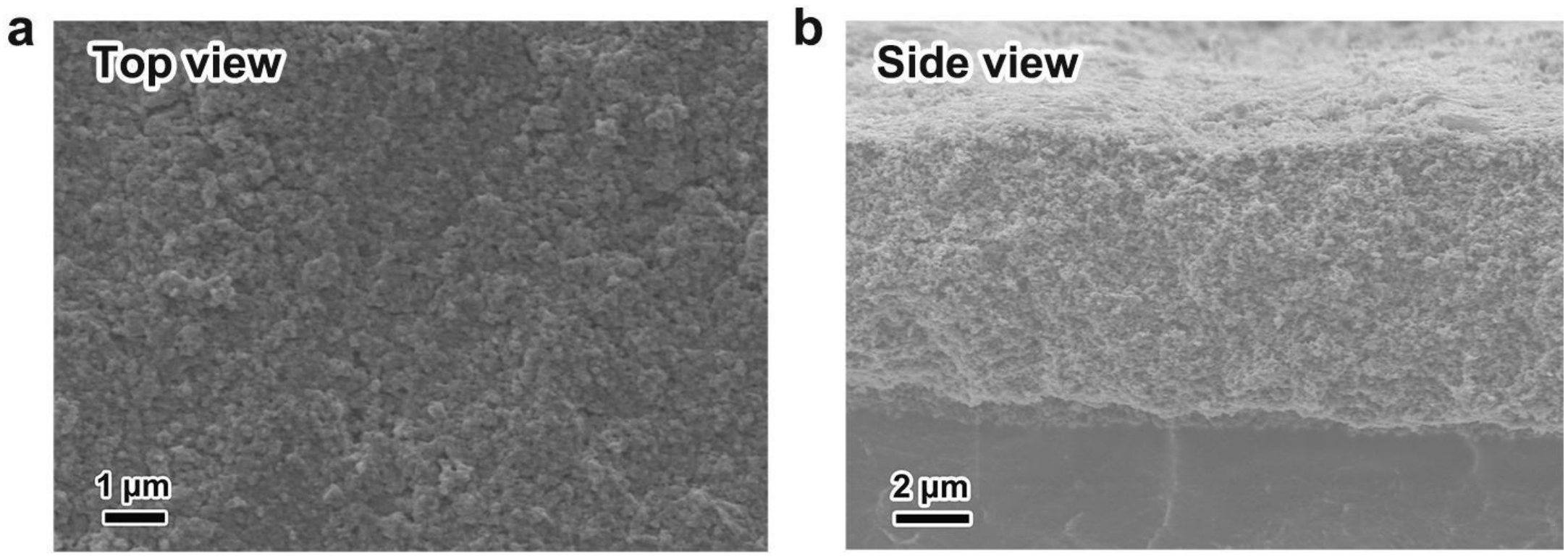

Supplementary Fig. 18 (a) Top view and (b) cross-sectional view of SEM images of the catalytic layer fabricated *via* the sequential spray strategy. The catalyst layer is fabricated with in-house synthesized IrO_2 at an iridium loading of 1 mg_{Ir} cm^{-2}.

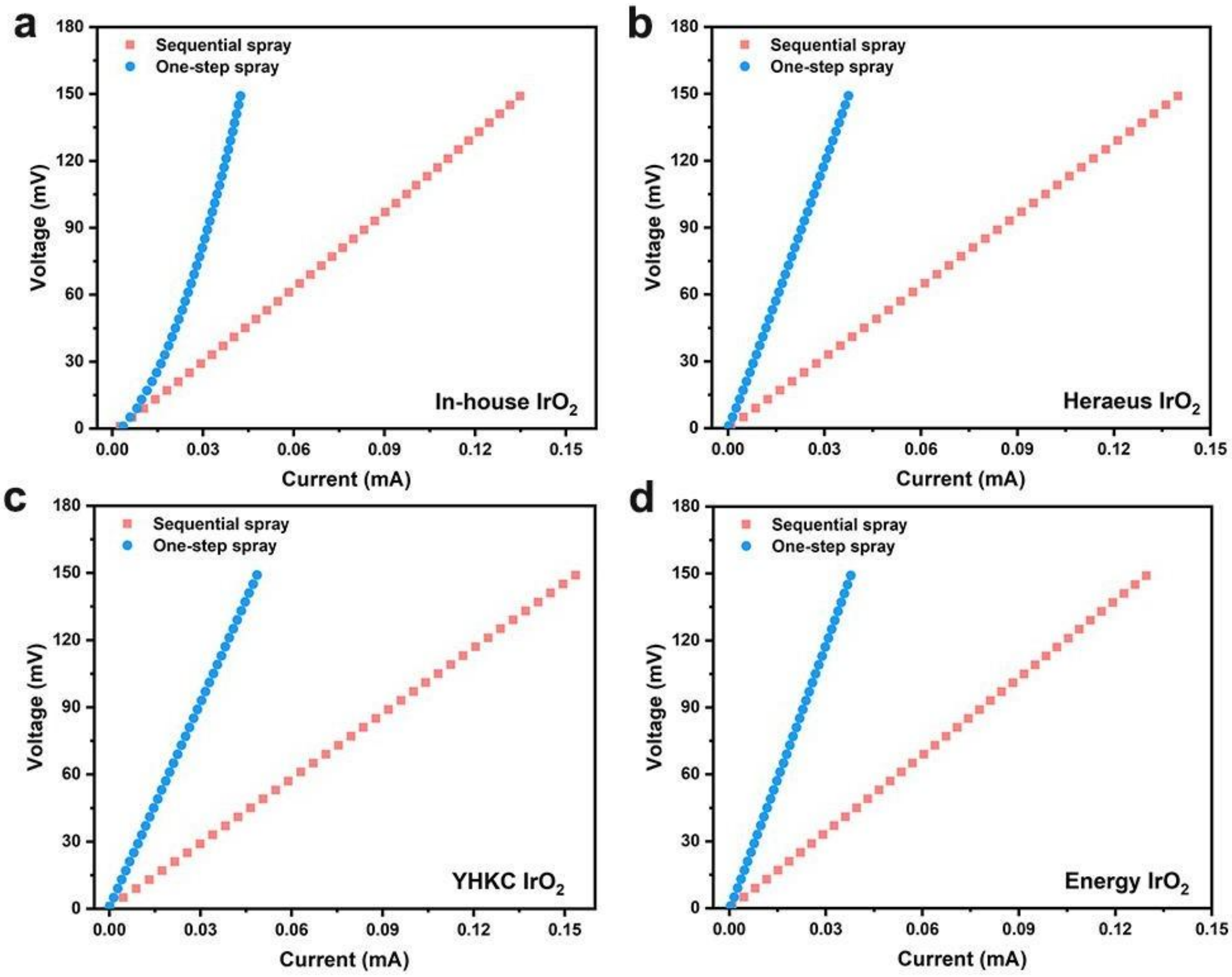

Supplementary Fig. 19 Comparison of the in-plane electronic conductivity of sequentially sprayed layers and one-step sprayed layers based on four different IrO_2 catalysts, including (a) an in-house synthesized material and three commercial catalysts from (b) Heraeus, (c) YHKC, and (d) Energy Chemical.

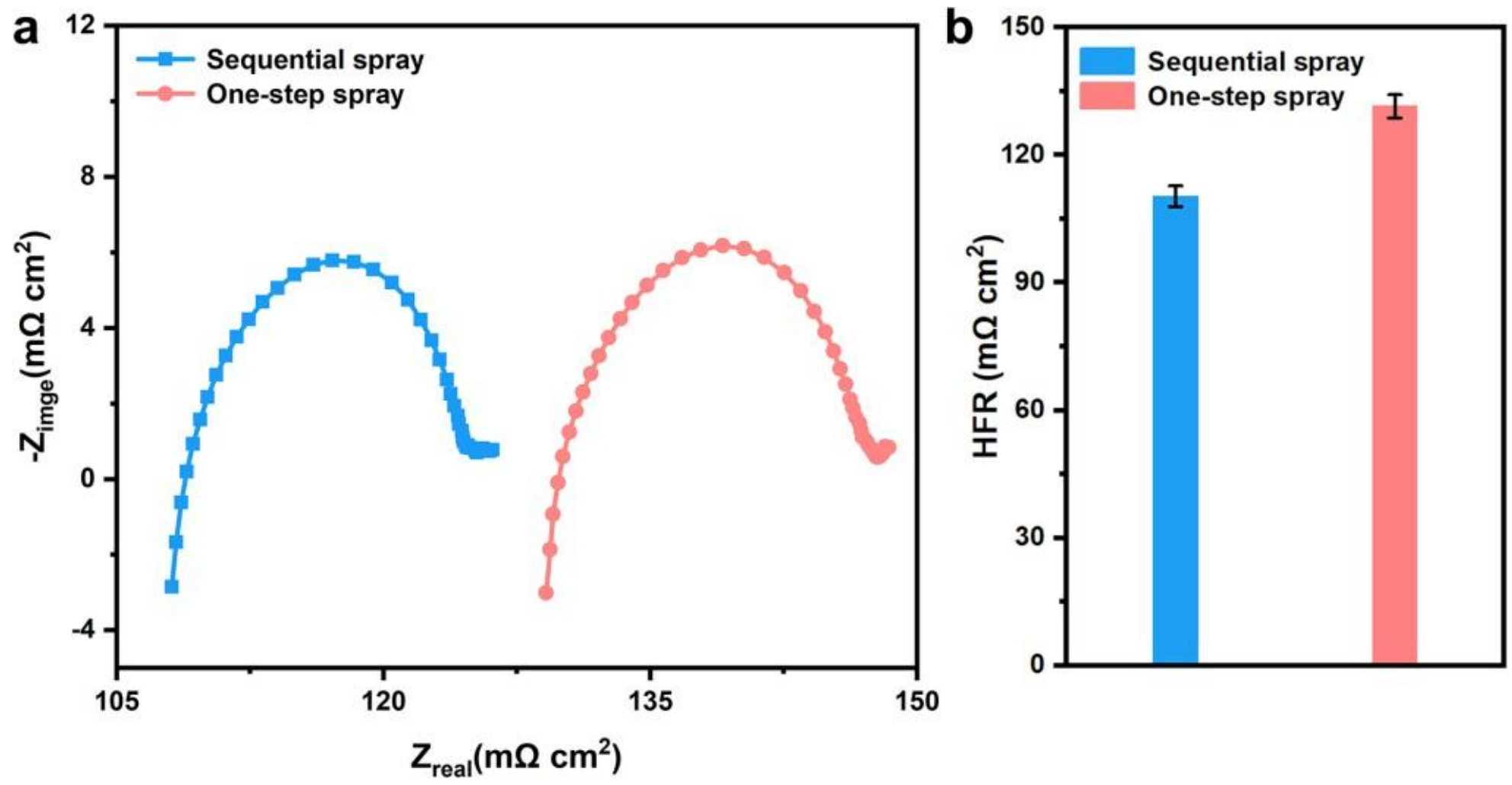

Supplementary Fig. 20 (a) Electrochemical impedance spectroscopy (EIS) spectra and (b) corresponding high-frequency resistance (HFR) values of PEMWE cells employing sequentially sprayed and one-step sprayed anode catalyst layers.

The HFR reflects the total ohmic resistance of the cell, including contributions from the membrane, catalyst layers, porous transport layers, bipolar plates, and their interfaces. All cell components were identical in our comparison except for the anode catalyst layer fabrication method. The PEMWE employing the sequentially sprayed anode exhibited an HFR of 110.3±2.4 mΩ cm^2, which is significantly lower than the 131.4±2.7 mΩ cm^2 measured for the conventional one-step sprayed anode. Since the anode catalyst layer structure is the only variable, this lower HFR directly confirms that the enhanced electronic connectivity established by the sequential spray strategy is maintained under fully hydrated operating conditions.

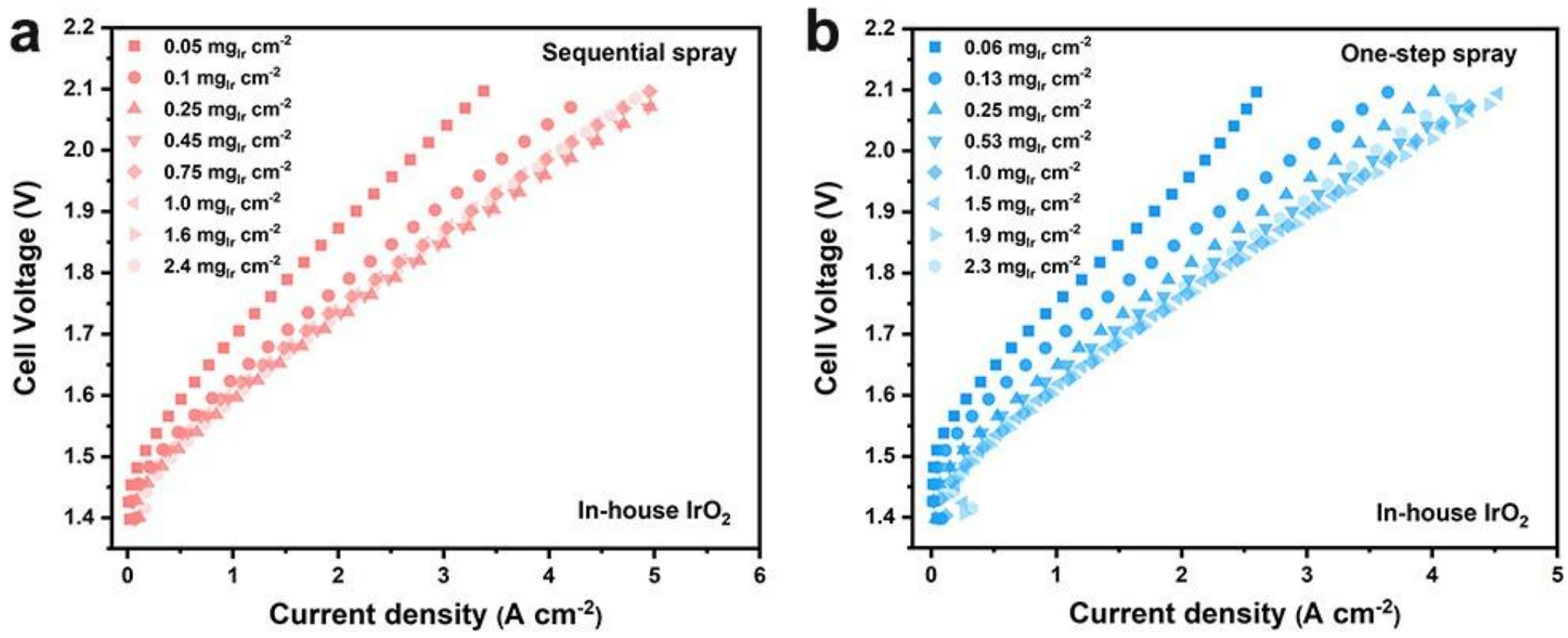

Supplementary Fig. 21 Steady-state polarization curves of PEMWE without iR-corrected using (a) sequentially sprayed layer and (b) one-step sprayed layer as anodes with different Ir loadings (80 °C, Nafion 115).

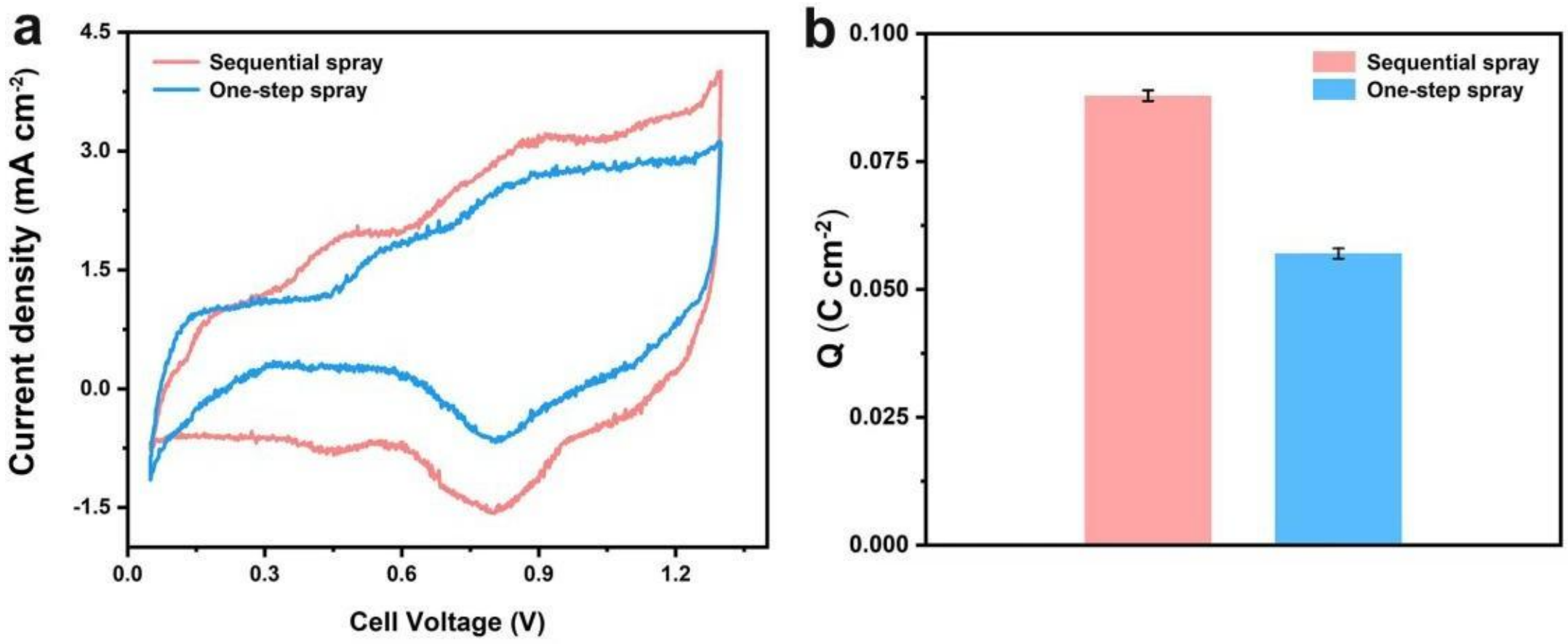

Supplementary Fig. 22 (a) Cyclic voltammetry (CV) curves for sequentially sprayed anode layer and one-step sprayed anode layer. (b) The pseudocapacitive charge (Q) values for sequentially sprayed anode layer and one-step sprayed anode layer calculated from CV curves. The catalyst layer is fabricated with in-house synthesized IrO_2 at an iridium loading of 1 mg_{Ir} cm^{-2}.

The sequentially sprayed anode layer exhibits a 36.7% larger CV area compared to the one-step sprayed anode layer. This result is consistent with the observed increase in activity, suggesting that the enhanced exposure of active sites due to the reactivation of electrochemically inactive zones is the primary factor for the improved activity.

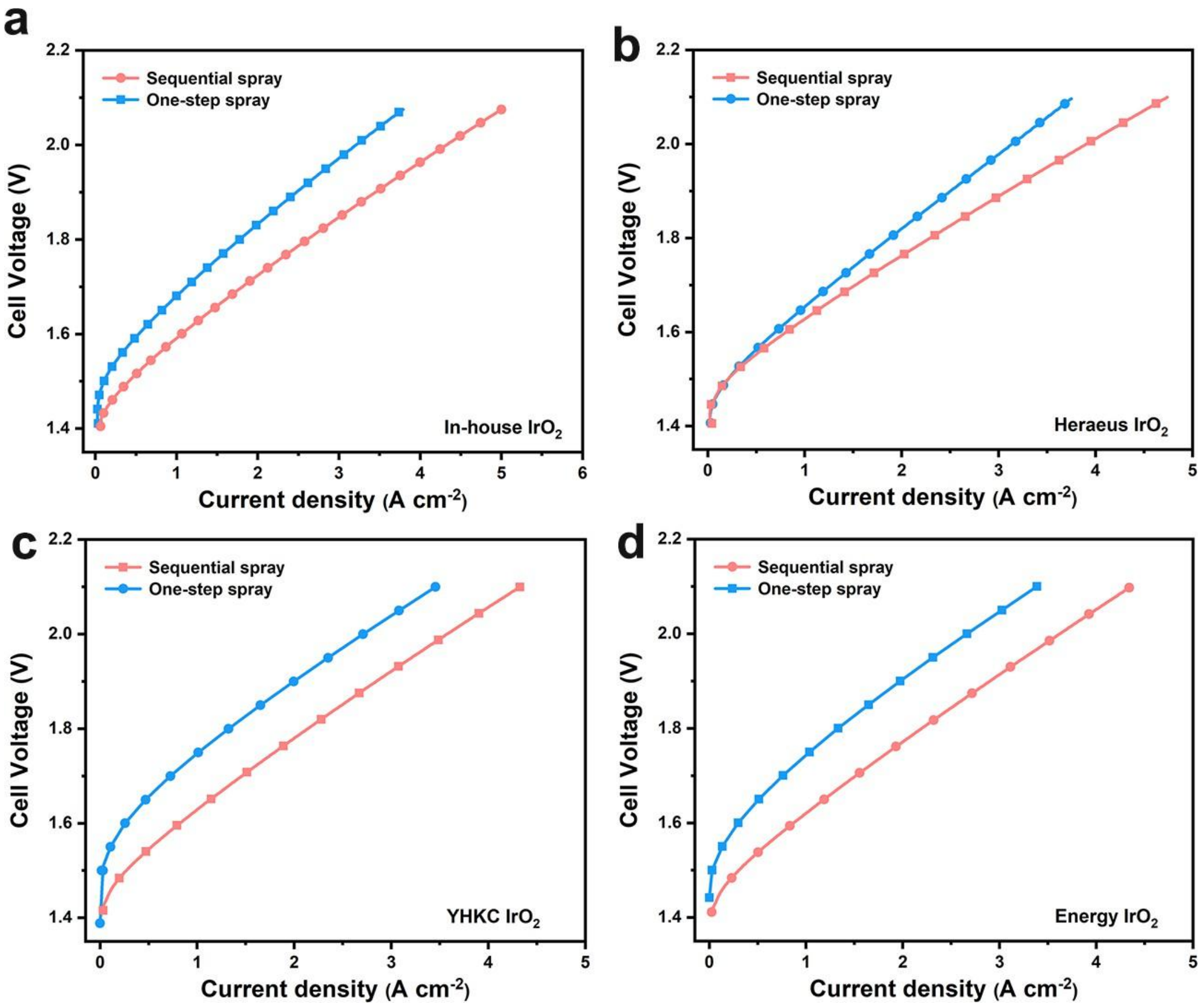

Supplementary Fig. 23 Steady-state polarization curves of PEMWE using sequentially sprayed layer and one-step sprayed layer as anodes (80 °C, Nafion 115, 0.25 mg_{Ir} cm^{-2}) based on four different IrO_2 catalysts, including (a) an in-house synthesized material and three commercial catalysts from (b) Heraeus, (c) YHKC, and (d) Energy Chemical.

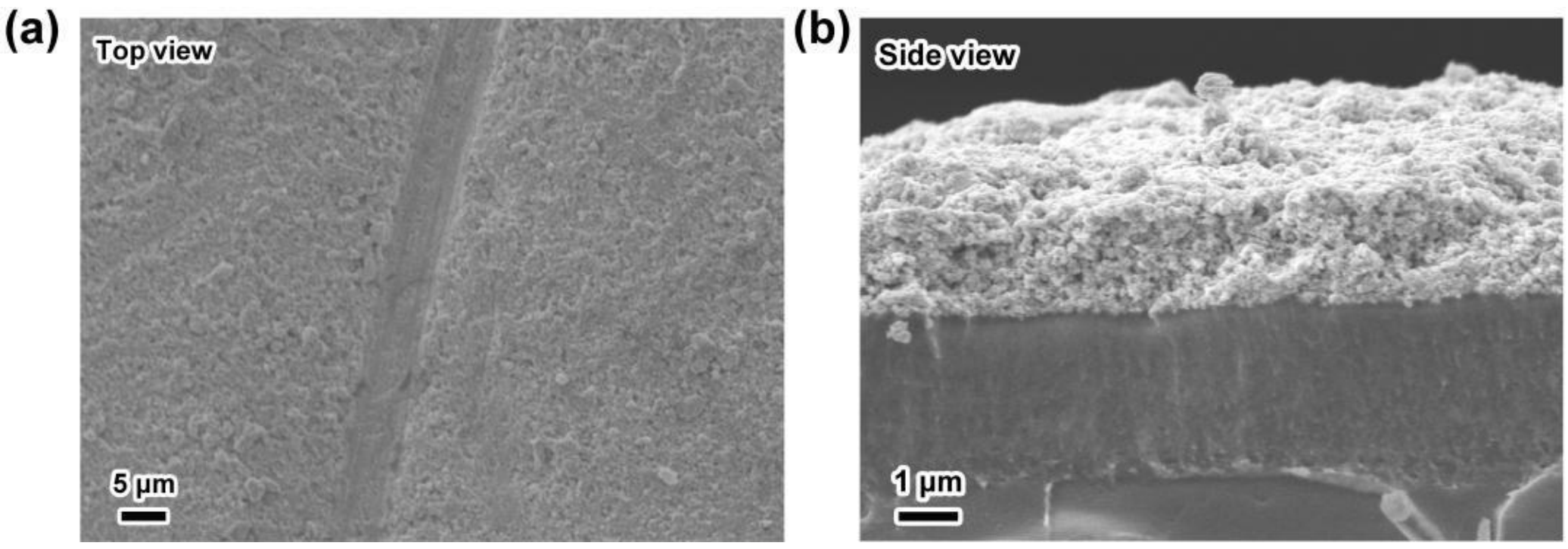

Supplementary Fig. 24 (a) Top-view and (b) side view SEM images of sequentially sprayed layer after long-term operation (120 hours at 2 A cm^{-2}).

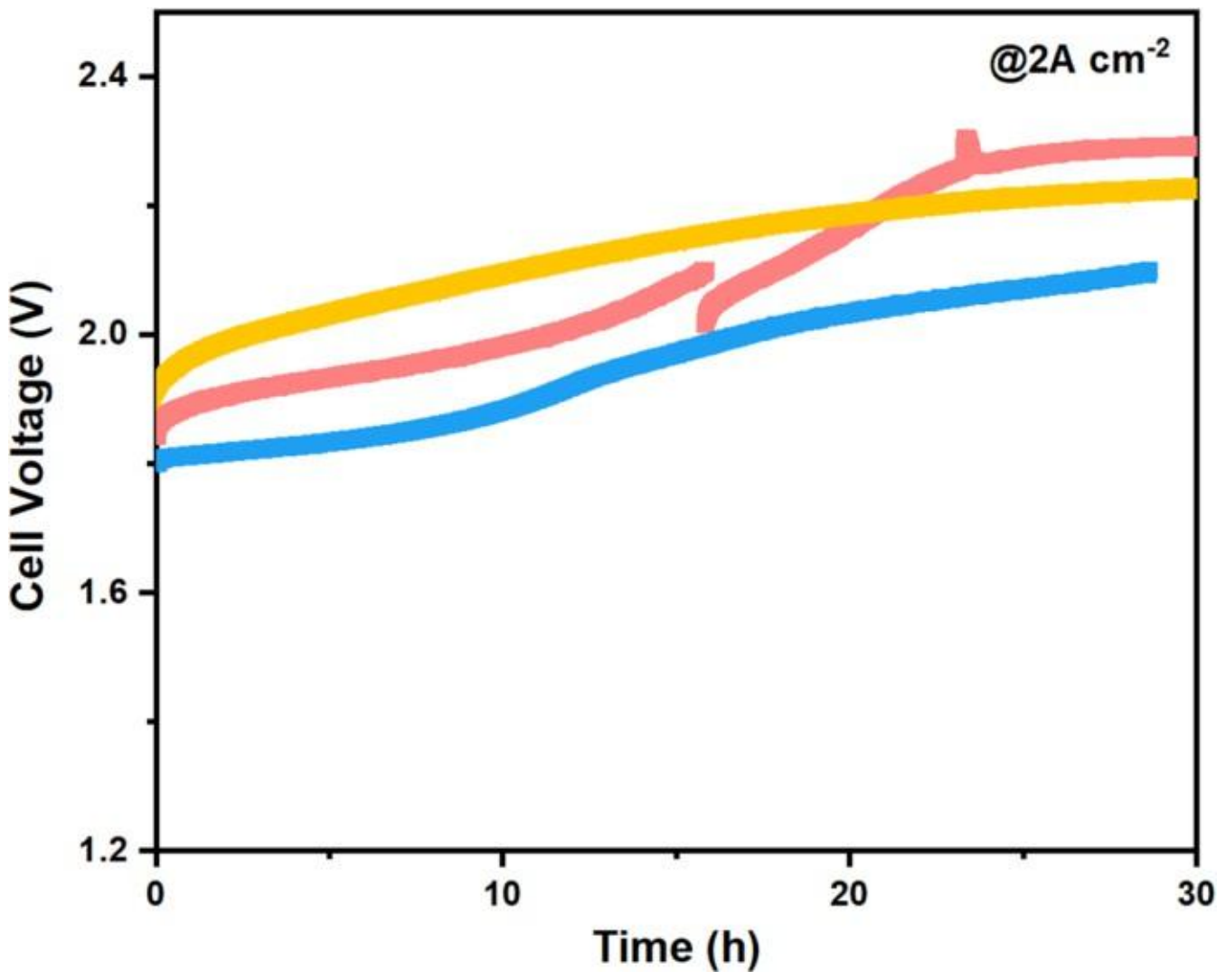

Supplementary Fig. 25 Chronopotentiometry curves of PEMWEs using one-step sprayed layer as anode under the Ir loading of 0.25 mg_{Ir} cm^{-2} at 1 A cm^{-2} current densities. Three independent replicate tests are shown, confirming the reproducibility of the rapid degradation behavior.

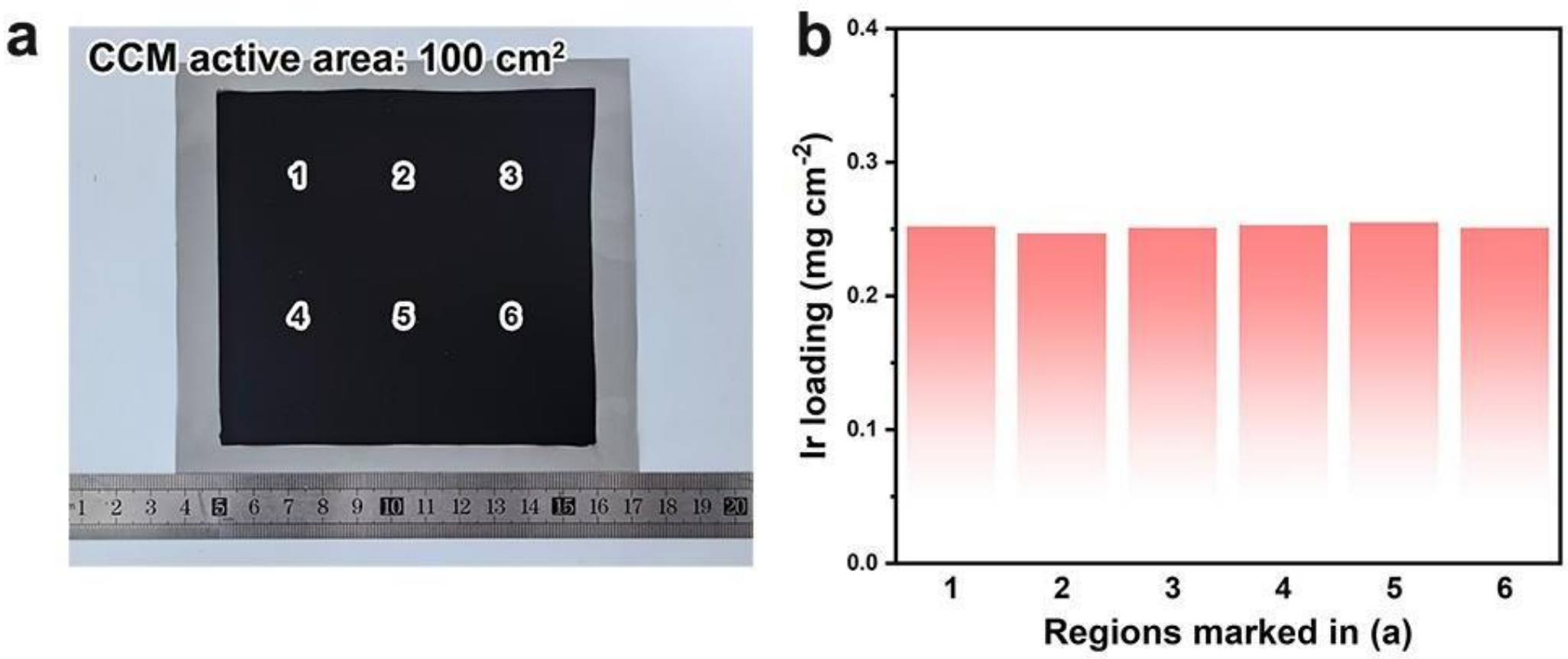

Supplementary Fig. 26 (a) Optical photograph of a large-area catalyst-coated membrane with an active area of 100 cm^2. (b) Determination of the iridium loading by XRF for different regions in (a).

It is clear that the entire membrane surface is homogeneously coated by the in-house synthesized IrO_2 catalyst.

Table S1 The three commercial grades of iridium oxide.

Materials	Specification	Ir mass ratio (wt%)
Heraeus IrO_2	99.99% metals basis	≥79.8
YHKC IrO_2	99.99% metals basis	≥80
Energy IrO_2	99.99% metals basis	≥85.5

Table S2 The four types of commercial ionomer specifications.

Type	Ionomer mass ratio (wt%)	Acid exchange capacity (EW)
DuPont D520	5	1000
DuPont D521	5	1100
DY DH3105	5	800
DY DH2205	5	900
