# Supplementary material for: Activating in-plane dead zones of anode catalyst layers in proton exchange membrane water electrolyzers
Source: Nat Commun. 2026 Jul 1;17:8211. doi: 10.1038/s41467-026-75223-1 (PMC13462438; doi:10.1038/s41467-026-75223-1)
Supplement: Supplementary file 3 [file 41467_2026_75223_MOESM2_ESM.pdf]

Description of Additional Supplementary Files

File Name: Supplementary Data 1

Description: Supplementary Data 1 contains all structures from molecular simulation. Atomic coordinates were provided in two text file formats (.gen and .xyz). The former contains the initial and final structures as well as the simulation box dimensions, while the latter contains the complete simulation trajectory.

File Name: Supplementary Video 1

Description: The high-speed and microscale visualization of gas bubble evolution occurring within the microchannel of PEMWEs employing different catalyst types.

File Name: Supplementary Video 2

Description: The high-speed and microscale visualization of gas bubble evolution occurring within the microchannel of PEMWEs with different catalyst loadings.

File Name: Supplementary Video 3

Description: The high-speed and microscale visualization of gas bubble evolution occurring within the microchannel of PEMWEs employing different ionomer types.

File Name: Supplementary Video 4

Description: The high-speed and microscale visualization of gas bubble evolution occurring within the microchannel of PEMWEs under different current densities.

File Name: Supplementary Video 5

Description: The high-speed and microscale visualization of gas bubble evolution occurring within the microchannel of PEMWEs under different clamping pressures.

File Name: Supplementary Video 6

Description: The high-speed and microscale visualization of gas bubble evolution occurring within the microchannel of PEMWEs with different ionomer/catalyst ratios.

File Name: Supplementary Video 7

Description: The high-speed and microscale visualization of gas bubble evolution occurring within the microchannel of PEMWEs employing different sequentially sprayed layers at a current density of 2 A cm^{-2}.
