# Supplementary material for: Activating in-plane dead zones of anode catalyst layers in proton exchange membrane water electrolyzers
Source: Nat Commun. 2026 Jul 1;17:8211. doi: 10.1038/s41467-026-75223-1 (PMC13462438; doi:10.1038/s41467-026-75223-1)
Supplement: Supplementary file 4 [file 41467_2026_75223_MOESM3_ESM.zip › Supplementary_Data_1/md_strus/Supplementary_Data_1_legend.docx]

**Supplementary_Data_1** contains all structures from molecular simulation. Atomic coordinates were provided in two text file formats (.gen and .xyz). The former contains the initial and final structures as well as the simulation box dimensions, while the latter contains the complete simulation trajectory.

The folder is organized as follows:

│

**├── pre-equilibration**	# Structures of pre-equilibration stage

**│	├── first.gen**	# The first‑frame structure from the pre‑equilibration simulation

**│	├── final.gen**	# The last‑frame structure from the pre‑equilibration simulation

**│	└── trj.xyz**	# The simulation trajectory of the pre‑equilibration stage

│

**├── production**	# Structures of production stage

**│	├── first.gen**	# The first‑frame structure from the production simulation

**│	├── final.gen**	# The last‑frame structure from the production simulation

**│	└── trj.xyz**	# The simulation trajectory of the production stage
